# Supplementary material for: Human pathogenic bacteria on high-touch dry surfaces can be controlled by warming to human-skin temperature under moderate humidity
Source: PLoS One. 2023 Sep 20;18(9):e0291765. doi: 10.1371/journal.pone.0291765 (PMC10511134; doi:10.1371/journal.pone.0291765)
Supplement: S1 Table — “CFU” indicates the number of live bacteria on dry surfaces in hospitals, “ATP” shows the human-contact frequency for these surfaces. (PDF) [file pone.0291765.s001.pdf]

Table S1: Data sets reused from previous our paper [19]

Data set	Hospital	Ward	ATP/CFU (per spot)																		temperature (°C)			moisture (humidity)(%			#people		
			instillation preparation table (nurse station)		routine worktable (nurse station)		nurse wagon(mobile station in nurse area)		next to sink		locker (outside) for hospital inpatients (room with multiple beds)		overbed table (room with multiple beds)		bedhead stand (private room)		overbed table for hospital inpatient (room with private bed)		hospital entrance floor		nurse station	room with multiple beds	private room	nurse station	room with multiple beds	private room	patients	medical staffs	total people
#Number	Hospital	Ward	ATP	CFU	ATP	CFU	ATP	CFU	ATP	CFU	ATP	CFU	ATP	CFU	ATP	CFU	ATP	CFU	ATP	CFU	T1	T2	T3	M1	M2	M3	N1	N2	N3
1	H	O	146	0	59	1	33	0	98	8	99	2	152	1	89	1	91	2	525	7	29.4	27.9	29.4	26	26	26	33	9	42
2	H	O	28	2	71	7	54	1	24	1	60	34	95	5	59	8	59	3	246	6	28.4	27.5	28.2	45	33	33	37	11	48
3	H	O	86	2	137	2	46	0	80	17	52	10	59	13	87	1	64	0	458	14	23.6	24.8	24.6	39	36	33	37	10	47
4	H	O	26	1	128	2	23	1	44	6	69	5	76	3	60	7	195	4	706	13	22.8	24.3	20.9	38	33	43	37	9	46
5	H	O	22	3	250	6	57	2	105	6	27	4	157	7	128	4	143	1	1010	11	22.2	24.2	25.9	38	28	28	45	9	54
6	H	O	63	0	657	4	72	1	39	4	228	37	483	11	47	3	2066	3	1504	11	22.1	23.9	24.1	39	33	30	43	9	52
7	H	O	37	1	231	3	143	6	55	2	154	1	659	1	139	1	155	1	965	14	23.1	24.6	23.9	31	29	30	48	10	58
8	H	O	80	3	332	10	224	0	75	10	147	49	1207	12	203	8	61	1	377	9	27	26.6	25.8	26	25	26	36	9	45
9	H	S	21	0	171	3	309	0	21	19	308	5	192	0	53	6	66	6	209	36	30.3	29.5	29.6	28	27	28	49	17	66
10	H	S	43	0	164	101	257	1	35	54	37	23	351	6	37	11	40	2	172	9	27.8	27.3	29	33	41	34	48	18	66
11	H	S	57	1	82	0	1028	3	70	47	103	3	143	11	240	10	691	41	537	63	23.4	22.6	21.5	51	51	49	49	18	67
12	H	S	34	0	134	6	38	0	48	2	104	3	398	7	102	6	227	2	1468	20	24.9	24	23.7	32	31	30	53	22	75
13	H	S	89	0	200	5	91	4	177	6	113	13	287	1	150	18	2110	30	1000	16	24.3	21.1	23.2	43	48	45	50	19	69
14	H	S	187	0	519	5	79	2	438	21	101	6	664	0	974	13	160	5	1956	11	24.3	22	23.2	30	34	31	49	23	72
15	H	S	141	0	153	2	49	2	150	2	90	6	274	0	183	16	349	1	1592	12	23.6	22.1	21.3	39	40	42	51	18	69
16	H	S	36	0	274	1	19	2	118	86	263	33	435	120	505	6	426	16	194	16	21.8	24.6	25.1	36	32	30	52	19	71
17	H	I	302	0	134	2	59	2	128	3	59	0	108	5	444	8	228	6	569	9	27.3	27.2	26.7	33	32	32	54	17	71
18	H	I	25	1	42	1	23	3	100	11	198	9	205	14	493	34	115	12	270	3	24.4	23.8	22.9	46	52	53	46	18	64
19	H	I	44	0	74	2	69	0	79	0	85	6	248	4	171	5	318	9	109	12	25.3	25	24.1	36	36	35	48	19	67
20	H	I	26	1	62	0	24	4	28	3	1044	13	35	8	133	4	315	2	176	4	25.6	25.8	25.5	38	49	49	42	16	58
21	H	I	69	0	129	6	121	10	144	3	475	32	121	9	236	5	59	12	431	10	25.7	26	24.2	32	29	25	47	19	66
22	H	I	27	1	64	1	133	6	402	16	474	15	210	12	1104	42	1570	66	1230	8	25.8	23.9	22.3	37	35	39	48	17	65
23	H	I	34	0	871	4	62	0	79	1	1129	8	1557	4	228	4	703	10	541	14	24.6	25.5	23.8	39	38	43	50	17	67
24	H	I	34	1	225	2	50	5	244	23	1429	10	392	26	85	7	296	11	115	31	25.9	26.7	27	33	32	32	49	18	67
25	K	I	24	1	196	1	87	2	91	6	201	9	499	1	1011	10	91	1	195	26	22.9	23.6	20.8	44	41	49	34	12	46
26	K	I	35	0	70	0	82	0	89	3	311	5	361	40	125	3	517	4	231	27	25	25.3	25.6	38	42	41	32	12	44
27	K	I	244	0	106	5	80	0	104	9	123	8	2892	25	249	4	1135	35	181	2	25.7	25.7	25.6	31	35	31	18	11	29
28	K	I	26	0	2767	2	111	1	42	4	1023	16	1119	8	224	1	117	8	159	11	26.2	26	26	35	35	34	31	12	43
29	K	I	72	0	208	3	130	0	115	1	165	13	110	3	112	2	473	34	1267	17	26.9	26.3	27	32	31	29	36	12	48
30	K	I	78	0	691	7	89	0	105	104	462	9	196	7	51	11	117	4	230	13	24.2	22	23	28	37	32	38	13	51
31	K	I	39	0	313	0	90	2	16	13	917	10	286	2	350	3	260	61	241	2	25.2	24.5	25.2	25	25	24	32	8	40
32	K	I	30	0	201	1	60	0	31	0	343	15	1336	5	296	5	43	0	187	5	24.6	24.7	24.3	36	41	36	39	12	51
33	K	S	147	0	130	3	165	0	68	103	117	3	63	3	174	8	267	2	288	10	24.2	23.8	22.8	33	37	38	35	14	49
34	K	S	40	1	57	1	73	1	297	93	109	3	38	1	70	9	567	3	1003	10	28.3	26.9	27.5	29	45	28	32	12	44
35	K	S	94	0	273	2	248	2	57	192	168	2	389	2	315	169	168	7	951	5	26.4	27	27.8	36	32	28	38	11	49
36	K	S	90	0	77	2	237	2	101	7	201	26	307	168	351	10	918	22	359	21	26.6	27.3	26.3	29	32	32	35	14	49
37	K	S	121	1	233	4	67	0	56	25	61	1	321	4	85	102	503	7	187	9	25	27	27.3	30	38	36	38	13	51
38	K	S	43	0	109	2	262	0	43	83	158	1	79	4	225	0	754	3	719	8	24.8	26.1	27	30	47	36	41	13	54
39	K	S	503	0	166	1	70	2	12	18	80	22	118	3	187	6	1900	4	269	6	26.4	26.4	26.4	24	25	25	38	12	50
40	K	S	52	0	60	1	337	1	201	1	67	66	121	20	229	6	265	25	740	6	26.6	26.8	26.7	37	40	38	38	12	50
41	K	O	140	0	206	0	122	1	90	12	158	0	241	3	100	3	156	1	542	1	27.3	25.7	25.7	24	30	27	37	17	54
42	K	O	36	1	60	5	93	0	81	3	253	4	552	2	84	4	141	12	436	20	28.5	28	28	24	26	26	31	16	47
43	K	O	127	1	192	1	105	1	57	7	653	7	190	1	112	12	449	9	421	5	29.6	27.3	26.2	24	33	28	32	18	50
44	K	O	60	0	66	0	98	0	25	3	294	19	598	0	118	4	656	0	413	12	27	27.2	26.8	26	25	23	33	14	47
45	K	O	121	0	250	3	181	3	58	85	69	1	679	4	201	5	140	1	453	2	26.5	27.4	27.4	26	38	32	36	15	51
46	K	O	111	1	531	6	160	2	39	7	748	4	354	3	372	5	51	0	262	11	27.4	27.2	27.2	26	26	23	42	14	56
47	K	O	113	1	501	2	102	0	26	2	98	7	443	6	262	7	30	3	391	5	28.1	27.3	27.1	22	23	21	43	17	60
48	K	O	34	0	77	0	37	1	28	1	31	8	1325	13	214	37	1043	9	343	6	26.7	26.8	28.4	34	34	36	39	14	53
49	M	I	88	1	81	46	47	4	51	3	47	4	46	24	146	28	98	3	227	16	26.8	23.5	25.8	35	40	36	21	10	31
50	M	I	110	1	51	15	89	0	49	14	147	19	547	18	107	1	122	9	930	11	21.3	21.1	20.3	45	47	46	22	10	32
51	M	I	60	58	47	151	42	2	31	120	na	200	247	32	269	8	203	3	245	3	24.1	24.7	24.8	30	33	30	24	9	33
52	M	I	86	1	396	9	98	30	39	5	363	340	354	22	339	13	472	2	606	8	23.1	23.6	23.4	27	26	26	25	11	36
53	M	I	208	6	190	8	292	5	58	35	312	7	534	29	277	1	613	3	1064	12	25.4	24.1	22.3	27	28	26	25	8	33
54	M	I	72	1	428	8	43	2	39	64	1827	1	473	20	113	1	454	4	1007	6	22.7	23.9	23.2	28	32	28	25	11	36
55	M	I	256	21	93	3	295	5	169	28	1439	25	250	0	606	8	106	13	933	21	23.4	21.4	23.3	31	32	26	23	12	35
56	M	I	106	0	532	1	310	10	169	39	816	9	117	14	111	7	114	2	482	7	26.7	26.4	26	25	23	23	24	9	33
57	M	I	64	na	339	na	74	na	65	na	293	na	76	na	2283	na	60	na	875	na	21.3	20.5	20.6	32	30	30	22	9	31
58	M	S	26	0	21	0	75	2	61	4	48	6	67	0	106	5	63	24	304	20	28.8	28.4	28.3	30	29	28	25	10	35
59	M	S	43	1	75	4	35	1	87	5	459	5	168	7	54	1	293	1	529	13	25.8	24.8	24.6	37	37	28	19	11	30
60	M	S	24	2	22	2	19	4	15	6	53	9	76	4	104	7	34	4	534	41	25.6	26.4	25.6	26	26	37	18	8	26
61	M	S	182	3	304	11	50	4	185	3	72	8	976	4	118	57	461	15	307	31	24.5	24.5	24.5	24	24	27	17	10	27
62	M	S	44	0	115	6	59	2	30	13	766	2	712	5	377	4	1735	6	845	114	25.8	25.4	25.1	25	26	23	14	8	22
63	M	S	38	2	84	2	148	3	77	1	522	7	670	4	3020	8	271	45	718	11	24.6	24.5	24.7	24	25	26	18	9	27
64	M	S	149	0	140	3	108	3	76	35	375	1	995	8	585	3	7887	9	960	12	23.5	24.6	24.3	25	23	24	21	10	31
65	M	S	75	1	280	0	84	9	248	38	295	16	1568	3	190	2	119	3	622	33	23.4	22.9	27.7	30	23	24	16	10	26
66	M	S	34	na	200	na	62	na	139	na	743	na	73	na	82	na	80	na	18	na	25.3	26.4	25.3	25	27	22	18	10	28

*I: Internal Medicine, S: Surgery, O: Obstetrics and Gynecology
*na: not avalable (no sampling)
