# Supplementary material for: Human pathogenic bacteria on high-touch dry surfaces can be controlled by warming to human-skin temperature under moderate humidity
Source: PLoS One. 2023 Sep 20;18(9):e0291765. doi: 10.1371/journal.pone.0291765 (PMC10511134; doi:10.1371/journal.pone.0291765)
Supplement: S2 Table — “Survival rate” indicates the value (number of live E. coli) for “T18” vs “T0.” (PDF) [file pone.0291765.s002.pdf]

Table S2: Data sets obtained from matrix analysis

Data set #number	temperature(°C)	humidity (%)	Survival rate
1	25	45	0.080519481
2	25	45	0.106493506
3	25	45	0.184415584
4	25	45	0.175324675
5	25	45	0.181818182
6	25	45	0.155844156
7	25	60	0.021818182
8	25	60	0.020779221
9	25	60	0.107012987
10	25	60	0.105974026
11	25	60	0.013506494
12	25	60	0.017402597
13	25	75	0.140833333
14	25	75	0.139166667
15	25	75	0.136666667
16	25	75	0.078333333
17	25	75	0.011666667
18	25	75	0
19	25	90	0.048
20	25	90	0.050666667
21	25	90	0.016
22	25	90	0.029333333
23	25	90	0.018666667
24	25	90	0.024
25	29	45	0.007700535
26	29	45	0.007700535
27	29	45	0.007058824
28	29	45	0.009304813
29	29	45	0.007058824
30	29	45	0.00802139
31	29	60	0.000355556
32	29	60	0.000651852
33	29	60	0.000414815
34	29	60	0.000533333
35	29	60	0.000171123
36	29	60	0.000171123
37	29	75	0.000117647
38	29	75	0.00015508
39	29	75	0.000117647
40	29	75	0.00013369
41	29	75	5.92593E-06
42	29	75	1.08642E-05
43	29	90	0.000969697
44	29	90	0.000969697
45	29	90	0.000787879
46	29	90	0.001090909
47	29	90	0.001939394
48	29	90	0.002242424
49	33	45	0.007202572
50	33	45	0.007588424
51	33	45	0.017363344
52	33	45	0.017106109
53	33	45	0.013054662
54	33	45	0.014405145
55	33	60	8.49858E-06
56	33	60	0
57	33	60	0
58	33	60	0
59	33	60	8.49858E-05
60	33	60	5.94901E-05
61	33	75	0.000154639
62	33	75	1.54639E-05
63	33	75	3.09278E-05
64	33	75	0
65	33	75	3.86598E-05
66	33	75	0.000811856
67	33	90	4.26396E-05
68	33	90	6.70051E-05
69	33	90	4.26396E-05
70	33	90	2.13198E-05
71	33	90	9.13706E-06
72	33	90	2.74112E-05
73	37	45	0.01212
74	37	45	0.01068
75	37	45	0.00684
76	37	45	0.00888
77	37	45	0.01116
78	37	45	0.012
79	37	60	0
80	37	60	0
81	37	60	0
82	37	60	0
83	37	60	0
84	37	60	5.52486E-06
85	37	75	1.06007E-05
86	37	75	0
87	37	75	0
88	37	75	0
89	37	75	0
90	37	75	0
91	37	90	0
92	37	90	0
93	37	90	0
94	37	90	0
95	37	90	0
96	37	90	0

*Survival rate: T18/T0
