# Supplementary figures and images for: Human pathogenic bacteria on high-touch dry surfaces can be controlled by warming to human-skin temperature under moderate humidity
Source: PLoS One. 2023 Sep 20;18(9):e0291765. doi: 10.1371/journal.pone.0291765 (PMC10511134; doi:10.1371/journal.pone.0291765)

Fig. S1

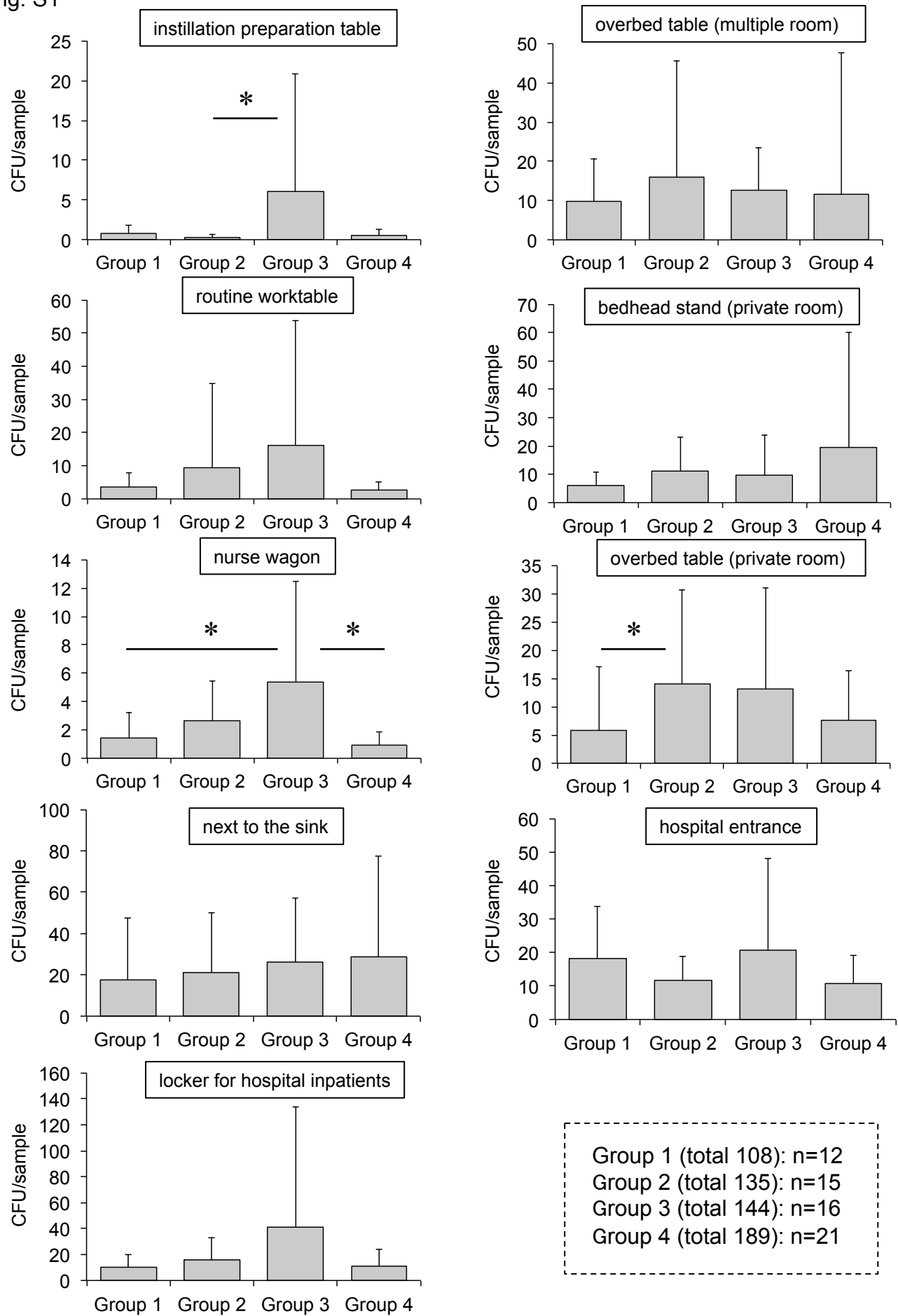

Supplement: S1 Fig — Group 1 was low temperature and high humidity [total 108: n = 12 (each bar)], group 2 was high temperature and high humidity [total 135: n = 15 (each bar)], group 3 was low temperature and low humidity [total 144: n = 16 (each bar)], and group 4 was high temperature and low humidity [total 21: n = 21 (each bar)]. See the detailed sampling sites in S1 Table. Bars show the average ± SD. *, p<0.05 with statistical significance between specific groups. (PDF) [file pone.0291765.s003.pdf]

Fig. S2

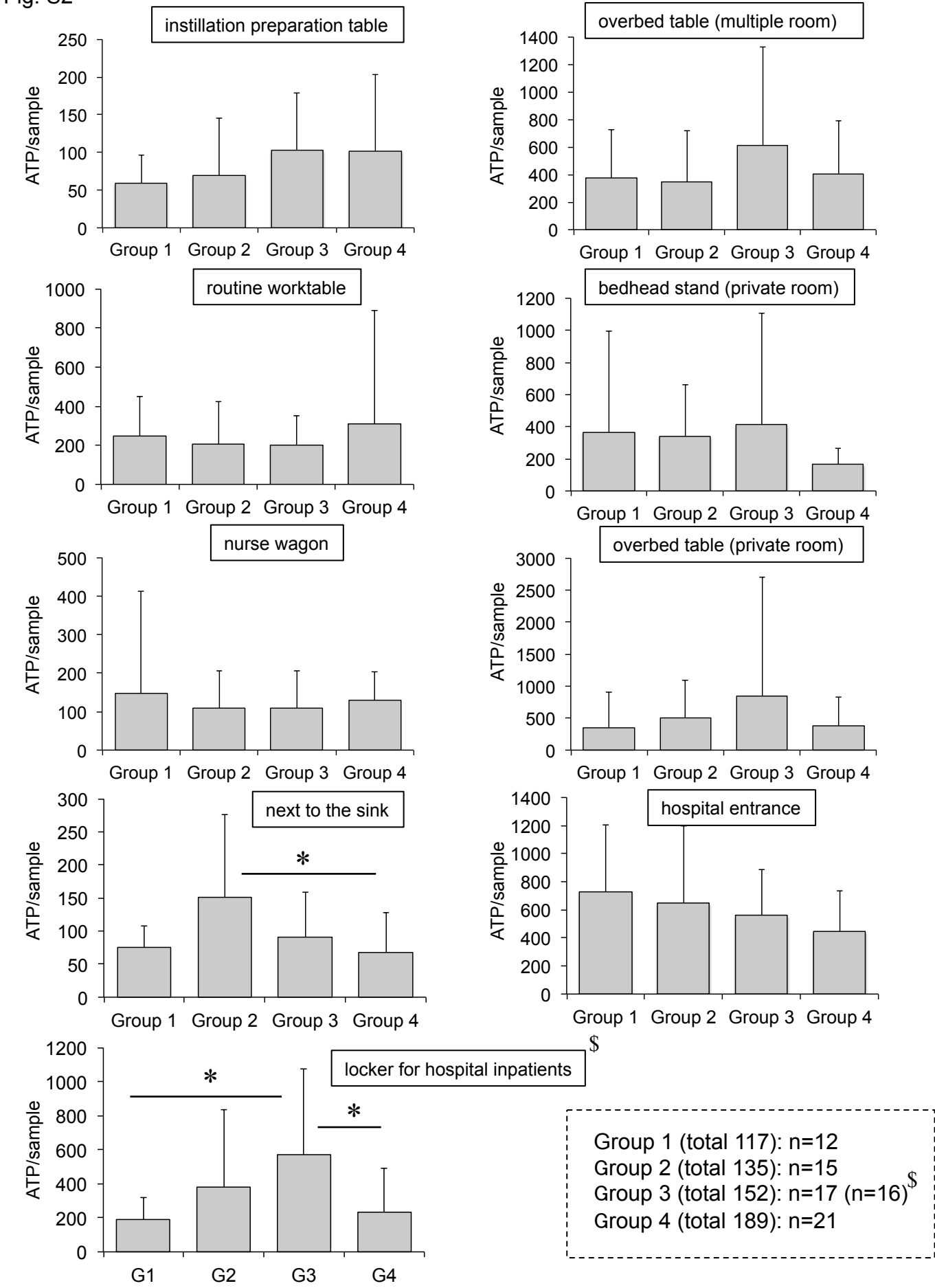

Supplement: S2 Fig — Group 1 was low temperature and high humidity, [total 117: n = 12 (each bar)], group 2 was high temperature and high humidity [total 135: n = 15 (each bar)], group 3 was low temperature and low humidity [total 152: n = 17 (each bar excluding “locker, n = 16”)], and group 4 was high temperature and low humidity [total 189: n = 21 (each bar)]. See the detailed sampling sites in S1 Table. Bars show the average ± SD. *, p<0.05 with statistical significance between specific groups. (PDF) [file pone.0291765.s004.pdf]

Fig. S3

A

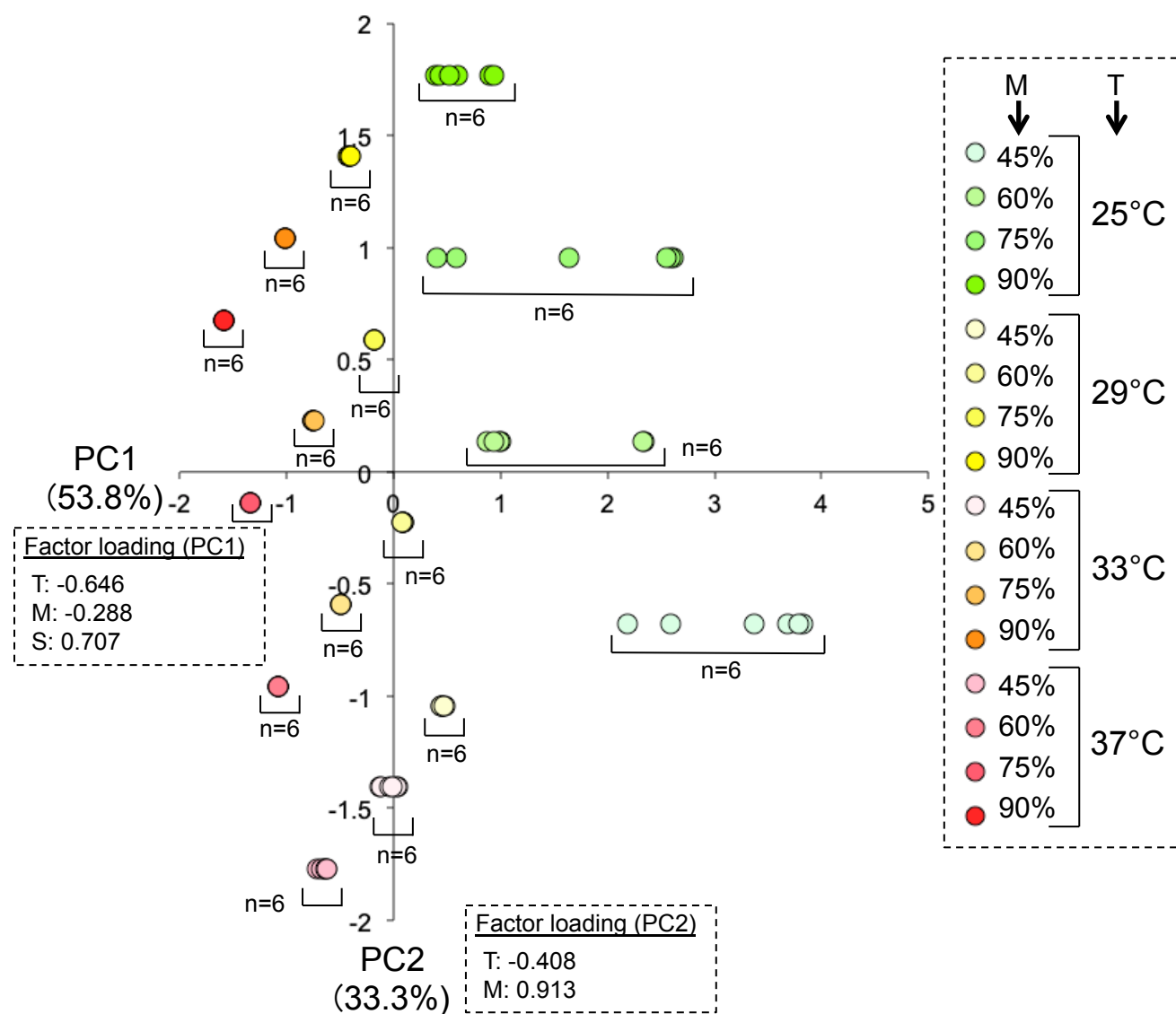

B

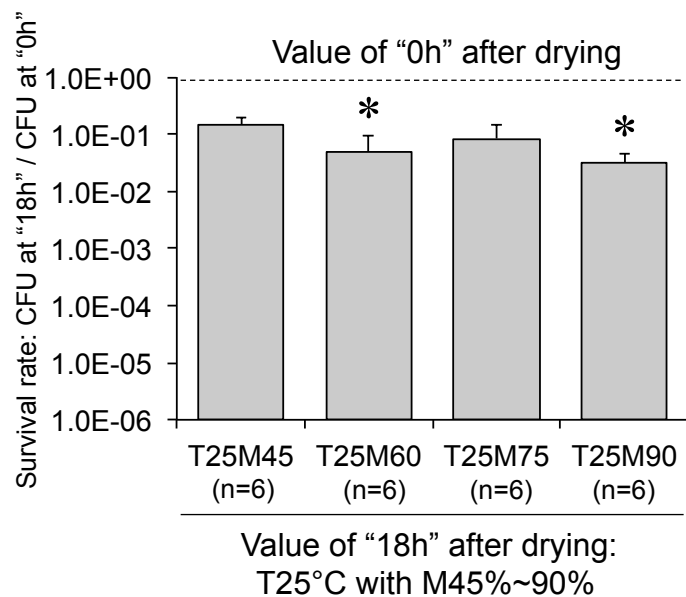

C

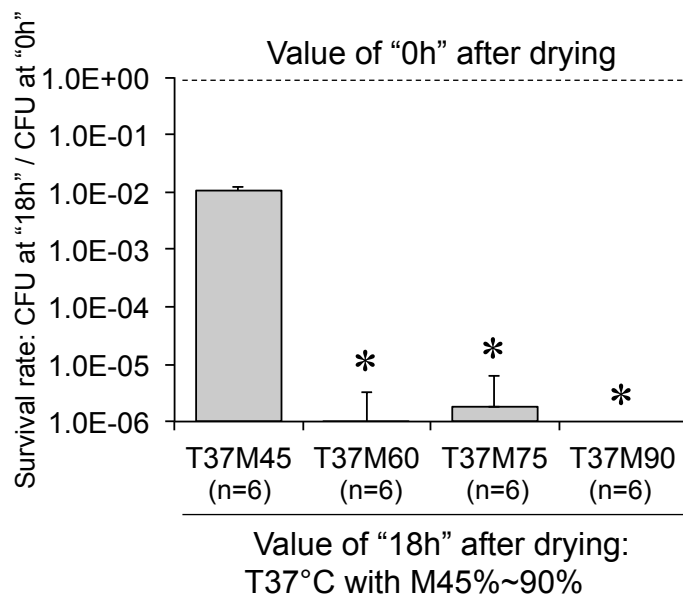

Supplement: S3 Fig — The experiment was performed in a thermo-hygrostat incubator. Various combinations of temperature (T) (25°C -37°C) and humidity (M) (45%-90%) were adjusted by the controller installed in the incubator. See the Materials and Methods. A. PCA scatter plot showing the impact on the survival rate of E. coil of temperature (25°C–37°C) and humidity (45%–90%). PC1-factor loading shows ˗0.646 (T: temperature), ˗0.288 (M: humidity), and 0.707 (S: the survival rate of E. coli). PC2-factor loading shows ˗0.408 (T) and 0.913 (M). Six runs were performed for each combination of matrices. B. Comparison of the survival rate of E. coli on dry surfaces (18 h/0 h) with 45%–90% humidity at 25°C. Bars (n = 6) show the average ± SD. *, p<0.05, with a statistically significant difference compared with the conditions for highest survival (T25/M45). C. Comparison of the survival rate of E. coli on dry surfaces (18 h/0 h) with 45%–90% humidity at 37°C. Bars (n = 6) show the average ± SD. *, p<0.05, with a statistically significant difference compared with the conditions for highest survival (T37/M45). (PDF) [file pone.0291765.s005.pdf]

Fig. S4

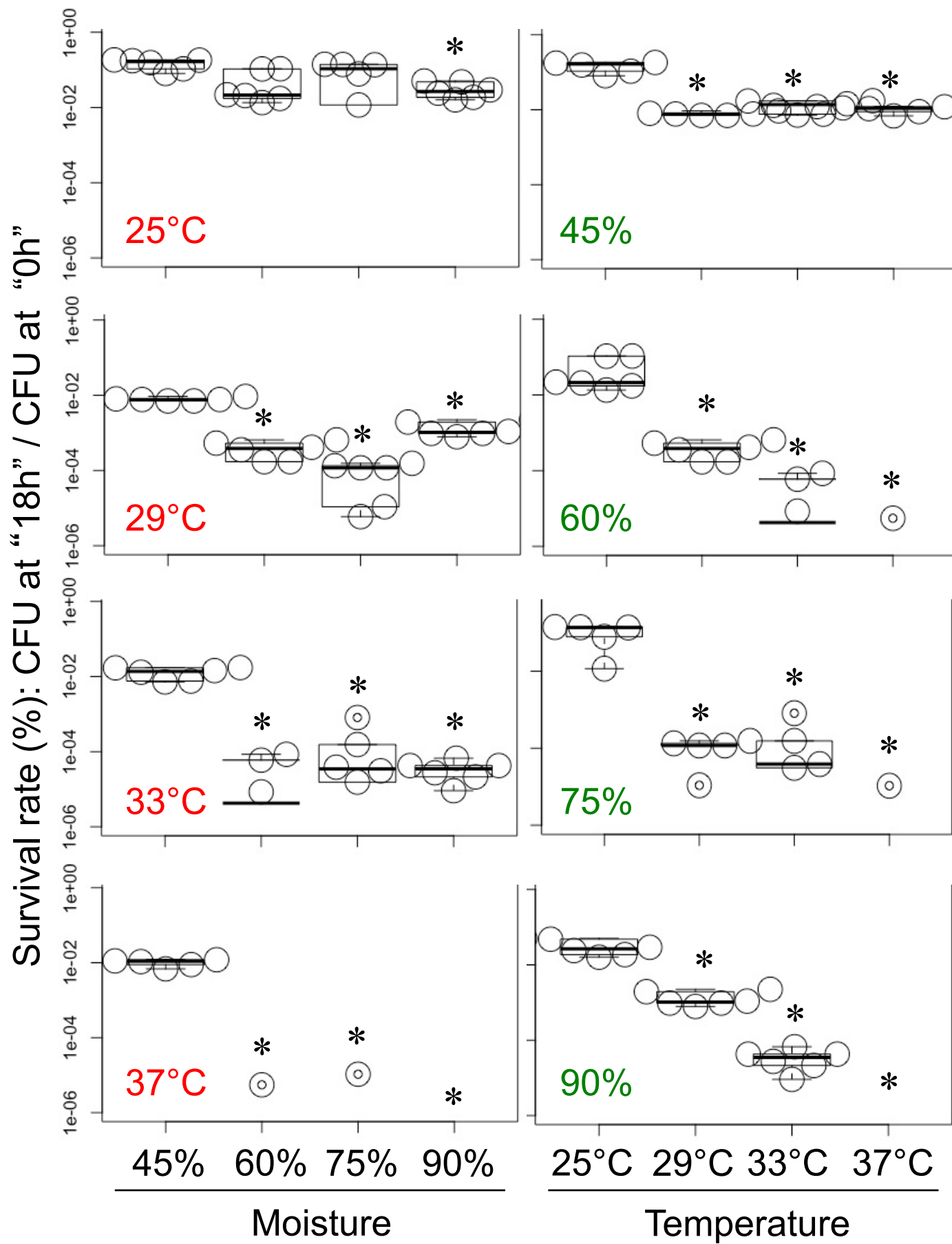

Supplement: S4 Fig — Bars (n = 6) show the average ± SD. *, p<0.05, with a statistically significant difference compared with the conditions for highest survival for each panel. (PDF) [file pone.0291765.s006.pdf]

A

—A —B —C

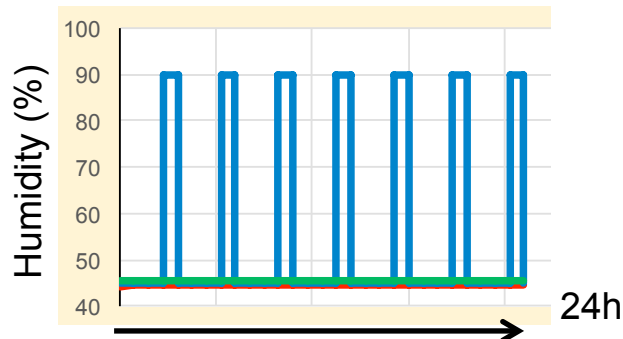

B

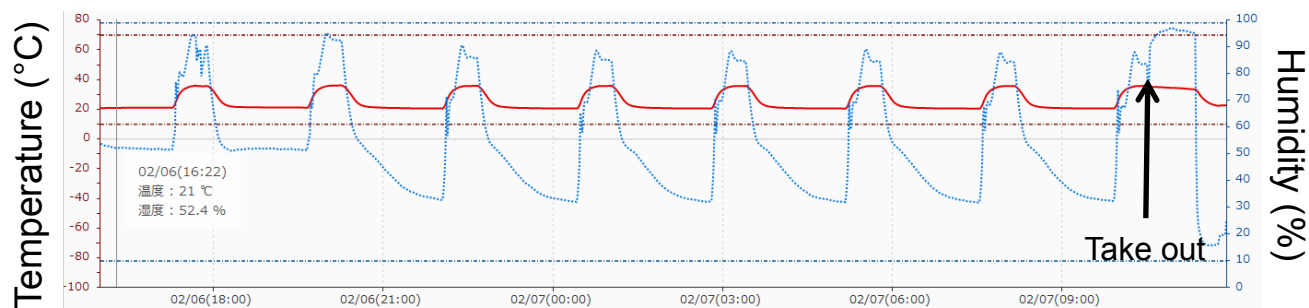

C

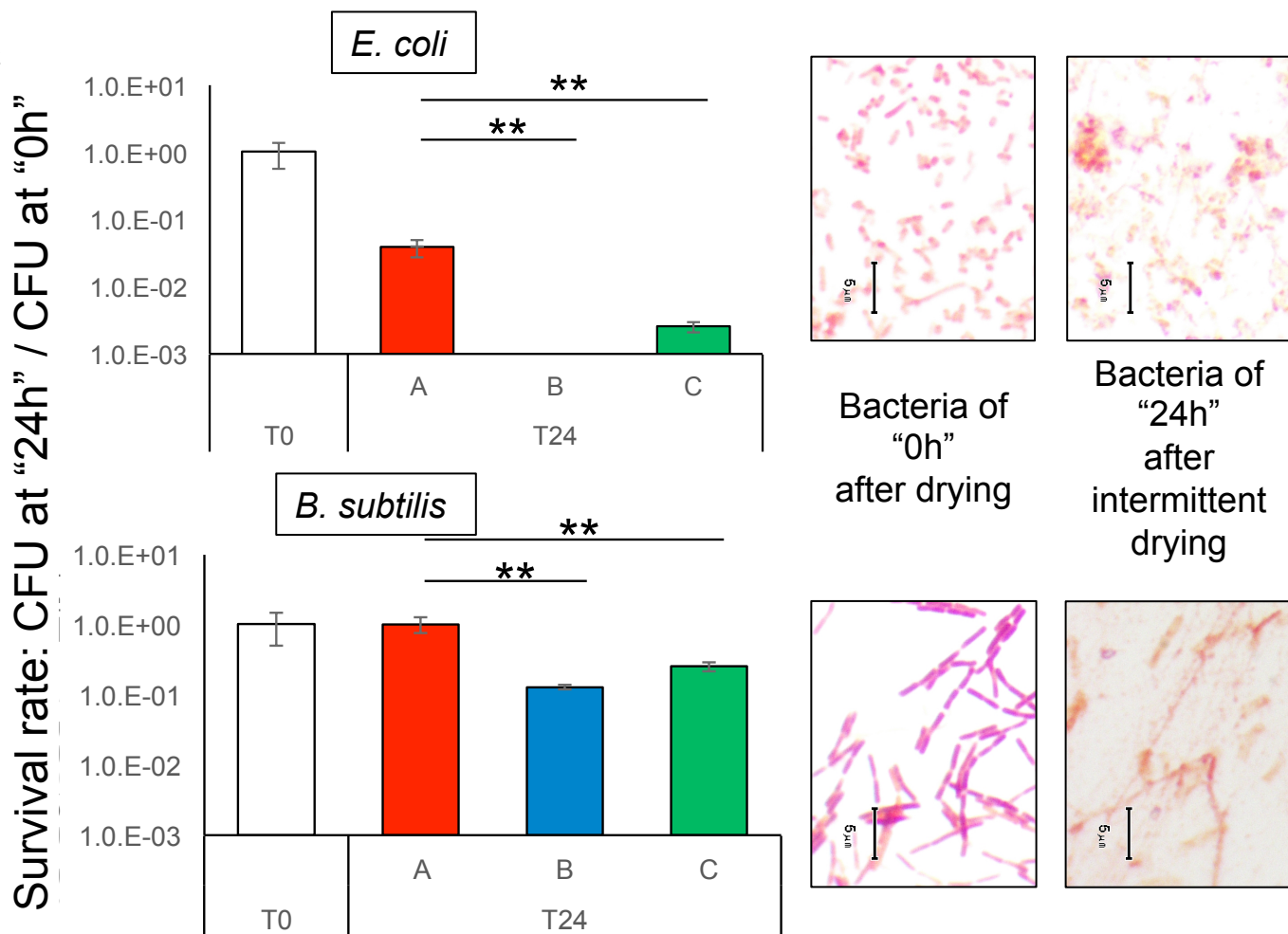

Supplement: S5 Fig — The experiment was performed in a thermo-hygrostat incubator. Cycle of temperature (22°C and 37°C) and humidity (RH: 45%-90%) were program-controlled by the controller installed in the incubator. See the Materials and Methods. A. Graphs show the schedule of intermittent warming and the temperature (left) and humidity (right) [blue: -B- (repeating 22°C with 45% humidity and 37°C with 90% humidity with an interval of 2 h)]. Fixed schedules were included as controls [red: -A- (22°C and 45%), green: -C- (37°C and 90%)]. B. Temperature and humidity monitoring with a data logger (AD-6324SET, AD Discover Precision, Tokyo, Japan). Red, temperature. Blue, humidity. Arrows indicate the timing of removal of the plate from the incubator. C. Effect of intermittent warming at human-skin temperature on decreasing the number of B. subtilis compared with E. coli. **, p<0.01, with a statistically significant difference compared with those of “red bar A.” (PDF) [file pone.0291765.s007.pdf]

Fig. S6

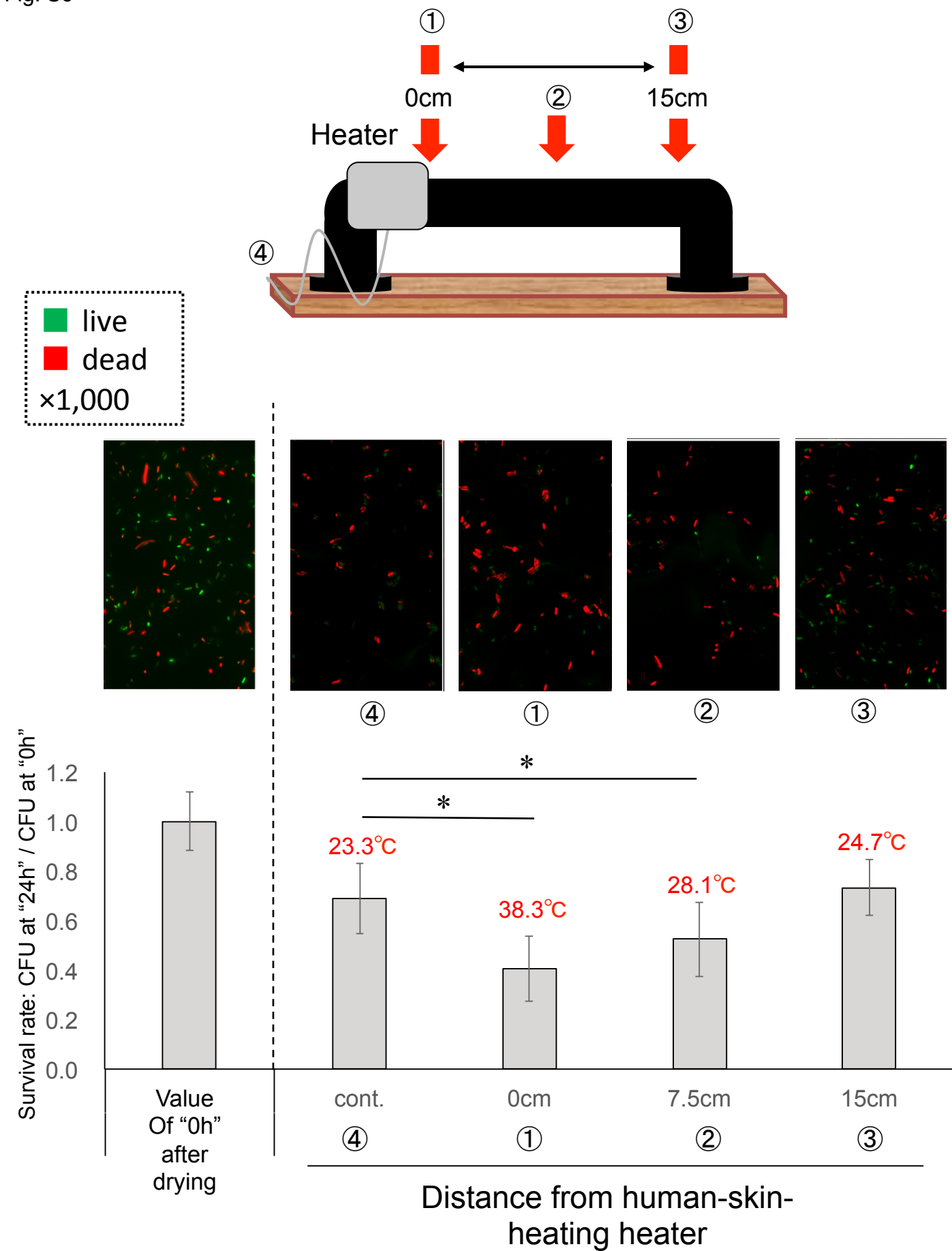

Supplement: S6 Fig — The experiment was performed in a thermo-hygrostat incubator with temperature 15°C and humidity 55%. Images show representative staining patterns (× 1,000). Area (red or green) was measured as the number of pixels by ImageJ software. Survival rate is shown as a ratio (T24/T0). Experiments were performed at least three times. Bars show the average ± SD. *, p<0.05, with a statistically significant difference compared with “cont.” (PDF) [file pone.0291765.s008.pdf]

Fig. S7

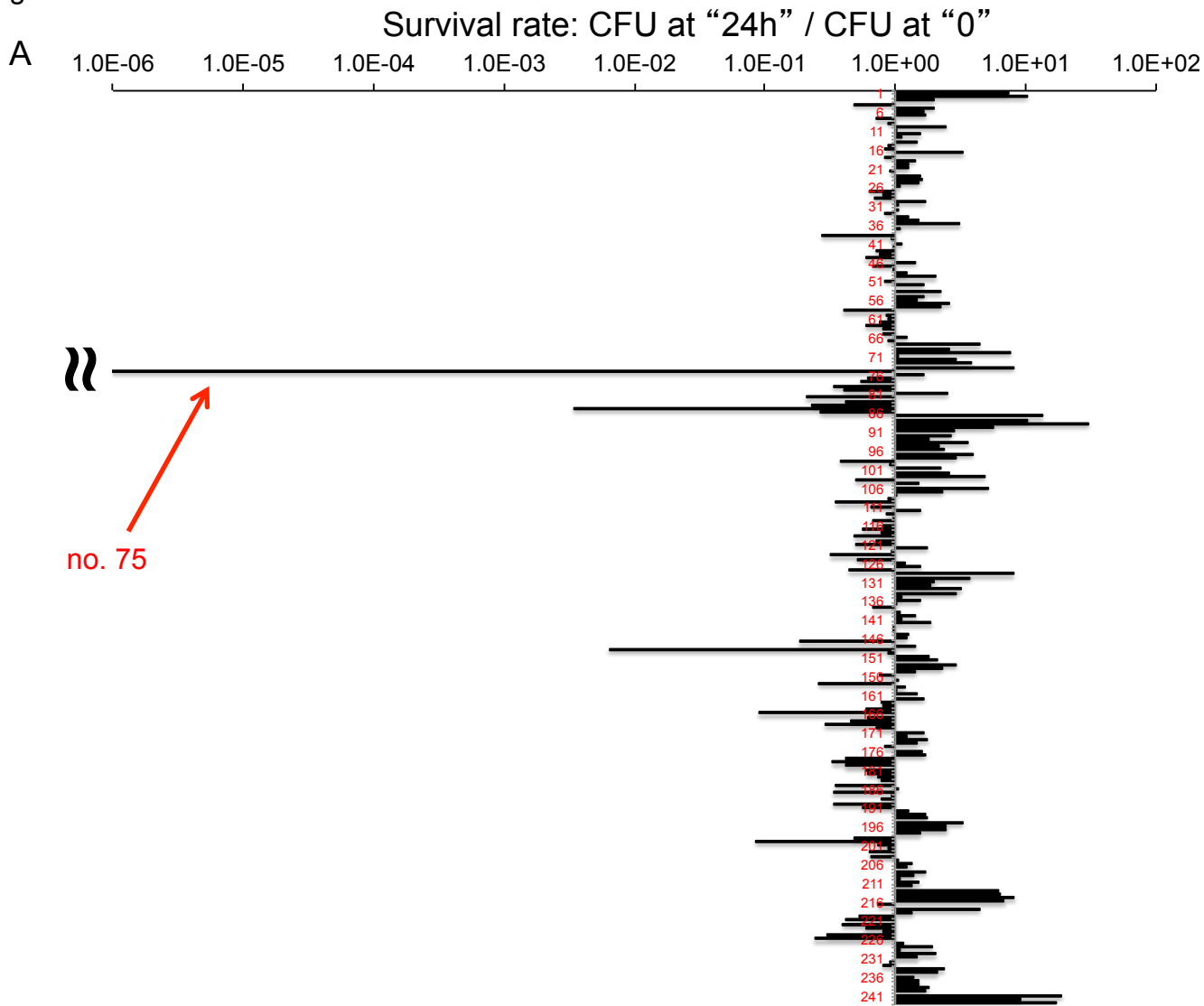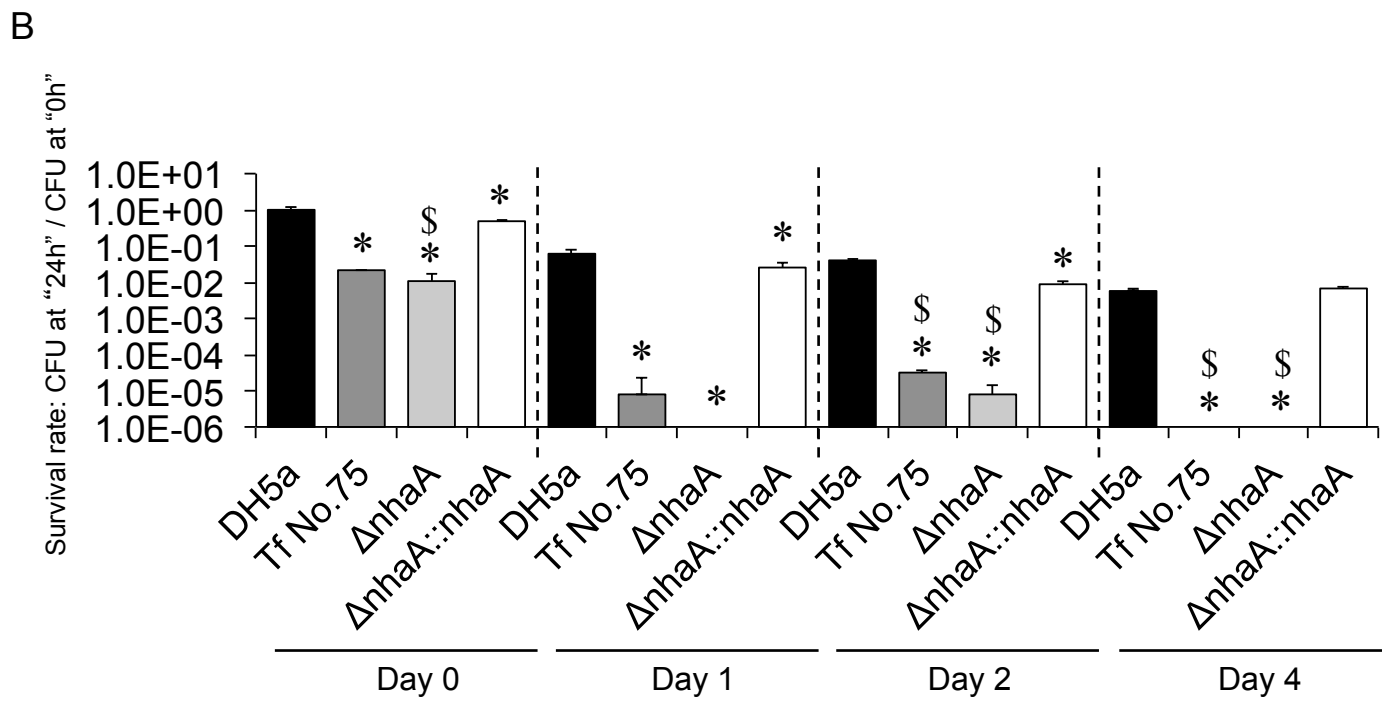

Supplement: S7 Fig — The experiment was performed at room temperature (approximately 22°C). A. The number of CFUs was compared between immediately-dried (T0) and 24-h incubated (T24) samples, and the results were expressed as a ratio (T24/T0). Red arrow indicates the mutant (no. 75) with the lowest resistance to desiccation among the library. B. Loss of dry resistance in the nhaA-disrupted strain and recovery in the reintroduced strain. The experiment was performed at least three times. Bars show the average ± SD. *, p<0.05, with a statistically significant difference compared with “E. coli DH5α” for each day. $, p<0.05, with a statistically significant difference compared with “E. coli DH5αΔnhaA::nhaA” for each day. (PDF) [file pone.0291765.s009.pdf]
