# Supplementary material for: Human pathogenic bacteria on high-touch dry surfaces can be controlled by warming to human-skin temperature under moderate humidity
Source: PLoS One. 2023 Sep 20;18(9):e0291765. doi: 10.1371/journal.pone.0291765 (PMC10511134; doi:10.1371/journal.pone.0291765)
Supplement: Attachment — Submitted filename: PONE-D-23-08650 (1).pdf [file pone.0291765.s010.pdf]

PLOS ONE

Human pathogenic bacteria on high-touched dry surfaces can be controlled by warming to human-skin temperature under moderate humidity

--Manuscript Draft--

**Manuscript Number:**	PONE-D-23-08650
**Article Type:**	Research Article
**Full Title:**	Human pathogenic bacteria on high-touched dry surfaces can be controlled by warming to human-skin temperature under moderate humidity
**Short Title:**	Pathogenic bacteria on dry surfaces warmed to human-skin temperature
**Corresponding Author:**	Hiroyuki Yamaguchi<br>Hokkaido University, Faculty of Health Sciences<br>Sapporo, Hokkaido JAPAN
**Keywords:**	Healthcare-associated infection; high-touched surface; dry surface; pathogenic bacteria; warming to human-skin temperature; humidity; principal component analysis; handrail
**Abstract:**	Healthcare-associated infections have become a major health issue worldwide. One route of transmission of pathogenic bacteria is through contact with “high-touch” dry surfaces, such as handrails. Regular cleaning of surfaces with disinfectant chemicals is ineffective against pathogenic bacteria and alternative control methods are therefore required. We previously showed that warming to human-skin temperature affected the survival of pathogenic bacteria on dry surfaces, but humidity was not considered in that study. Here, we investigated environmental factors that affect the number of live bacteria on dry surfaces in hospitals by principal component analysis of previously-collected data, and experimentally verified the effect of warming to human-skin temperature on the survival of pathogenic bacteria on dry surfaces under humidity control. The results revealed that dry surfaces at low temperature and low humidity had much higher bacterial counts. Experimentally, warming to human-skin temperature significantly suppressed the survival of pathogenic bacteria on dry surfaces, such as plastic surfaces or handrails, under moderate humidity. Furthermore, intermittent heating to human-skin temperature reduced the survival of spore-forming bacteria. NhaA, an Na+/H+ antiporter, was found to regulate the survival of bacteria on dry surfaces, and the inhibitor 2-aminoperimidine enhanced the effect of warming at human-skin temperature on the survival of pathogenic bacteria on dry surfaces. Thus, warming to human-skin temperature under moderate humidity is a useful method for impairing live pathogenic bacteria on high-touched surfaces, thereby helping to prevent the spread of healthcare-associated infections.
**Order of Authors:**	Ayano Konno
	Torahiko Okubo
	Yoshiaki Enoeda
	Tomoko Uno
	Toyotaka Sato
	Shin-ichi Yokota
	Rika Yanao
	Hiroyuki Yamaguchi
**Additional Information:**	
**Question**	**Response**
**Financial Disclosure**<br><br>Enter a financial disclosure statement that describes the sources of funding for the	This study was funded by a Grant-in-Aid for Scientific Research, KAKENHI (grant number: 20K20613) (to H.Y.). The funders had no role in the study design, data collection and analysis, decision to publish, or preparation of the manuscript.

Powered by Editorial Manager® and ProduXion Manager® from Aries Systems Corporation

work included in this submission. Review the submission guidelines for detailed requirements. View published research articles from *PLOS ONE* for specific examples.<br><br>This statement is required for submission and **will appear in the published article** if the submission is accepted. Please make sure it is accurate.<br><br>**Unfunded studies**<br>Enter: *The author(s) received no specific funding for this work.*<br><br>**Funded studies**<br>Enter a statement with the following details:<br>• Initials of the authors who received each award<br>• Grant numbers awarded to each author<br>• The full name of each funder<br>• URL of each funder website<br>• Did the sponsors or funders play any role in the study design, data collection and analysis, decision to publish, or preparation of the manuscript?<br>• **NO** - Include this sentence at the end of your statement: *The funders had no role in study design, data collection and analysis, decision to publish, or preparation of the manuscript.*<br>• **YES** - Specify the role(s) played.<br><br>* typeset	
**Competing Interests**<br><br>Use the instructions below to enter a competing interest statement for this submission. On behalf of all authors, disclose any competing interests that could be perceived to bias this work—acknowledging all financial support and any other relevant financial or non-financial competing interests.<br><br>This statement is required for submission and **will appear in the published article** if the submission is accepted. Please make sure it is accurate and that any funding sources listed in your Funding Information	The authors have declared that no competing interests exist.

Powered by Editorial Manager® and ProduXion Manager® from Aries Systems Corporation

later in the submission form are also declared in your Financial Disclosure statement.<br><br>View published research articles from *PLOS ONE* for specific examples.<br><br>**NO authors have competing interests**<br><br>Enter: *The authors have declared that no competing interests exist.*<br><br>**Authors with competing interests**<br><br>Enter competing interest details beginning with this statement:<br><br>*I have read the journal's policy and the authors of this manuscript have the following competing interests: [insert competing interests here]*<br><br>* typeset	
**Ethics Statement**<br><br>Enter an ethics statement for this submission. This statement is required if the study involved:<br><br>• Human participants<br>• Human specimens or tissue<br>• Vertebrate animals or cephalopods<br>• Vertebrate embryos or tissues<br>• Field research<br><br>Write "N/A" if the submission does not require an ethics statement.<br><br>General guidance is provided below. Consult the submission guidelines for detailed instructions. **Make sure that all information entered here is included in the Methods section of the manuscript.**	The ethics committees of each of the hospitals that participated in this study determined that there was no need for ethical approval for the acquisition of dry surface wipes and data relating to environmental factors (temperature, humidity, and the number of people at sampling sites) within the hospital. Before collecting samples or data, we explained the study design and informed consent was orally obtained from all medical staff and hospital inpatients intending to participate in this study. During sampling, we protected the privacy and confidentiality of personal information under the supervision of each of the hospital managers and in accordance with the Helsinki Declaration.

**Format for specific study types**<br><br>**Human Subject Research (involving human participants and/or tissue)**<br>• Give the name of the institutional review board or ethics committee that approved the study<br>• Include the approval number and/or a statement indicating approval of this research<br>• Indicate the form of consent obtained (written/oral) or the reason that consent was not obtained (e.g. the data were analyzed anonymously)<br><br>**Animal Research (involving vertebrate animals, embryos or tissues)**<br>• Provide the name of the Institutional Animal Care and Use Committee (IACUC) or other relevant ethics board that reviewed the study protocol, and indicate whether they approved this research or granted a formal waiver of ethical approval<br>• Include an approval number if one was obtained<br>• If the study involved *non-human primates*, add *additional details* about animal welfare and steps taken to ameliorate suffering<br>• If anesthesia, euthanasia, or any kind of animal sacrifice is part of the study, include briefly which substances and/or methods were applied<br><br>**Field Research**<br><br>Include the following details if this study involves the collection of plant, animal, or other materials from a natural setting:<br>• Field permit number<br>• Name of the institution or relevant body that granted permission	
**Data Availability**<br><br>Authors are required to make all data underlying the findings described fully available, without restriction, and from the time of publication. PLOS allows rare exceptions to address legal and ethical concerns. See the PLOS Data Policy and FAQ for detailed information.	Yes - all data are fully available without restriction

A Data Availability Statement describing where the data can be found is required at submission. Your answers to this question constitute the Data Availability Statement and **will be published in the article**, if accepted.<br><br>**Important:** Stating 'data available on request from the author' is not sufficient. If your data are only available upon request, select 'No' for the first question and explain your exceptional situation in the text box.<br><br>Do the authors confirm that all data underlying the findings described in their manuscript are fully available without restriction?	
**Describe where the data may be found in full sentences. If you are copying our sample text, replace any instances of XXX with the appropriate details.**<br><br>• If the data are **held or will be held in a public repository**, include URLs, accession numbers or DOIs. If this information will only be available after acceptance, indicate this by ticking the box below. For example: *All XXX files are available from the XXX database (accession number(s) XXX, XXX.*).<br>• If the data are all contained **within the manuscript and/or Supporting Information files**, enter the following: *All relevant data are within the manuscript and its Supporting Information files.*<br>• If neither of these applies but you are able to provide **details of access elsewhere**, with or without limitations, please do so. For example:<br><br>*Data cannot be shared publicly because of [XXX]. Data are available from the XXX Institutional Data Access / Ethics Committee (contact via XXX) for researchers who meet the criteria for access to confidential data.*<br><br>*The data underlying the results presented in the study are available from (include the name of the third party*	All relevant data are within the manuscript and its Supporting Information files.

and contact information or URL).<br>• This text is appropriate if the data are owned by a third party and authors do not have permission to share the data.<br><br>* typeset	
Additional data availability information:	

Manuscript Click here to access/download;Manuscript;Konno et al text 20230323.docx

Human pathogenic bacteria on high-touched dry surfaces can be controlled by warming to human-skin temperature under moderate humidity

Ayano Konno[a], Torahiko Okubo[a], Yoshiaki Enoeda[a], Tomoko Uno[b,c], Toyotaka Sato[d,e,f,g], Shin-ichi Yokota[d], Rika Yano[c], Hiroyuki Yamaguchi[a]*

[a]Department of Medical Laboratory Science, Faculty of Health Sciences, Hokkaido University, North-12, West-5, Kita-ku, Sapporo 060-0812, Japan

[b]Department of Nursing, Sapporo Medical University School of Medicine, South-1, West-17, Chuo-ku, Sapporo, 060-8556, Japan

[c]Department of Fundamental Nursing, Faculty of Health Sciences, Hokkaido University, North-12, West-5, Kita-ku, Sapporo 060-0812, Japan

[d]Department of Microbiology, Sapporo Medical University School of Medicine, South-1, West-17, Chuo-ku, Sapporo, 060-8556, Japan

[e]Laboratory of Veterinary Hygiene, Faculty of Veterinary Medicine, Hokkaido University, Sapporo, North-18, West-9, Kita-ku, 060-0818, Japan

[f]Graduate School of Infectious Diseases, Hokkaido University, North-18, West-9, Kita-ku, Sapporo, 060-0818, Japan

[g]One Health Research Center, Hokkaido University, North-18, West-9, Kita-ku, Sapporo,

060-0818, Japan

***Correspondence:** Hiroyuki Yamaguchi, Department of Medical Laboratory Science, Faculty of Health Sciences, Hokkaido University, North-12, West-5, Kita-ku, Sapporo 060-0812, Japan

Tel.: +81-11-706-3326; Fax: +81-11-706-3326

E-mail: hiroyuki@med.hokudai.ac.jp

Running title: Pathogenic bacteria on dry surfaces warmed to human-skin temperature

E-mail addresses:

Ayano Konno: 2525ayakon@gmail.com

Torahiko Okubo: t.okubo@hs.hokudai.ac.jp

Yoshiaki Enoeda: Yoshiaki.Enoeda@jp.ricoh.com

Tomoko Uno: tuno@sapmed.ac.jp

Toyotaka Sato: sato.t@vetmed.hokudai.ac.jp

Shin-ichi Yokota: syokota@sapmed.ac.jp

Rika Yanao: r-yano@med.hokudai.ac.jp

Hiroyuki Yamaguchi: hiroyuki@med.hokudai.ac.jp

Abstract

Healthcare-associated infections have become a major health issue worldwide. One route of transmission of pathogenic bacteria is through contact with "high-touch" dry surfaces, such as handrails. Regular cleaning of surfaces with disinfectant chemicals is ineffective against pathogenic bacteria and alternative control methods are therefore required. We previously showed that warming to human-skin temperature affected the survival of pathogenic bacteria on dry surfaces, but humidity was not considered in that study. Here, we investigated environmental factors that affect the number of live bacteria on dry surfaces in hospitals by principal component analysis of previously-collected data, and experimentally verified the effect of warming to human-skin temperature on the survival of pathogenic bacteria on dry surfaces under humidity control. The results revealed that dry surfaces at low temperature and low humidity had much higher bacterial counts. Experimentally, warming to human-skin temperature significantly suppressed the survival of pathogenic bacteria on dry surfaces, such as plastic surfaces or handrails, under moderate humidity. Furthermore, intermittent heating to human-skin temperature reduced the survival of spore-forming bacteria. NhaA, an Na^+/H^+ antiporter, was found to regulate the survival of bacteria on dry surfaces, and the inhibitor 2-aminoperimidine enhanced the effect of warming at human-skin temperature on the survival of pathogenic bacteria on dry surfaces. Thus, warming to human-skin temperature under moderate humidity is a useful method for impairing live pathogenic bacteria on high-touched surfaces, thereby helping to prevent the spread of healthcare-associated infections.

Keywords:

Healthcare-associated infection; high-touched surface; dry surface; pathogenic bacteria; warming to human-skin temperature; humidity; principal component analysis; handrail

Classification

BIOLOGICAL SCIENCES (Microbiology)

Abbreviations

HAIs: healthcare-associated infections; CFUs: colony-forming units; PCA: principal component analysis; PCs: principal components; NhaA: Na^+/H^+ antiporter A; 2-AP: 2-aminoperimidine.

Introduction

The occurrence and spread of healthcare-associated infections (HAIs) caused by human pathogenic bacteria in public medical facilities, including long-term care homes and nursing homes, has become a global problem [1-3]. Compared with community-acquired infections in the general population, HAIs often affect patients who are older and have weakened immune systems, making them more difficult to treat and often negatively impacting on the quality of life and prognosis of patients [4]. The World Health Organization reported that the spread of HAIs prolonged hospital stays, increasing the financial burden on healthcare systems and medical insurance premiums, as well as increasing the risk of emergence of resistant bacteria [5, 6]. For these reasons, there is an urgent need for new strategies to prevent HAIs.

One factor contributing to the spread of HAIs is contact between medical staff and patients, in particular, through frequently-touched dry surfaces, such as handrails and doorknobs, referred to as “high-touched surfaces” [7]. Such surfaces become hot spots for infection because human pathogenic bacteria adhere to them and survive for a long period of time [7]. Therefore, at present, as a preventive measure against HAIs, regular cleaning of dry surfaces using disinfectants is performed at hospitals. However, such cleaning procedures are costly and do not prevent outbreaks of microorganisms that are resistant to disinfectant. In fact, it has been reported that extended spectrum β-lactamase-producing *Escherichia coli* strains are resistant to multiple disinfectants [8] and *Acinetobacter* possesses a chlorhexidine efflux pump [9]. Furthermore,

Pseudomonas aeruginosa, a bacterium that causes opportunistic infections, reportedly acquires resistance to multiple antibiotics as a result of the persistence of disinfectants containing benzalkonium chloride on regularly cleaned surfaces [10]. It has also been reported that, despite routine cleaning for the management of HAIs, vancomycin-resistant enterococci and methicillin-resistant *Staphylococcus aureus* persist in the healthcare environment for days, threatening the spread of infection [11]. This is partly due to the overuse of antibiotics or disinfectants [12].

Biofilms formed on dry surfaces cannot be permeated by disinfectant, and bacteria continue to survive and cause a high frequency of infections in hospitals after cleaning [13]. Several studies have shown that the cleanliness of contact surfaces is inadequate in terms of preventing infection [14, 15]. New control measures therefore need to be developed to replace regular cleaning with disinfectants.

Dry surfaces are an unsuitable environment for bacteria to maintain vital metabolic activity [16], and bacteria have therefore developed strategies to resist drying. However, the survival mechanisms that bacteria employ in dry environments are not fully understood. Environmental factors such as temperature and humidity have been reported to contribute to the survival of bacteria in dry environments [16, 17]. In our previous studies, we clarified that temperature affected the population of airborne bacteria in an underground pedestrian space in Sapporo, Japan [18], as well as the number of live bacteria on dry surfaces in a hospital environment [19, 20]. Furthermore,

we reported preliminary evidence that the survival of pathogenic bacteria was significantly impaired on dry-plastic surfaces warmed to 37°C [21]. These findings strongly suggest that the number of live bacteria can be controlled on dry surfaces simply by adjusting the temperature.

Here, we extend these findings by investigating the dynamics of bacteria on dry surfaces in hospitals depending on changes in temperature and humidity, and the degree of human contact. We show experimentally that human pathogenic bacteria on dry surfaces such as handrails can be controlled by warming of the surfaces to human-skin temperature under moderate humidity. Furthermore, we demonstrate that NhaA, an Na^{+}/H^{+} antiporter, is a crucial factor in supporting the survival of bacteria under dry conditions, and that an inhibitor (2-aminoperimidine: 2-AP) of NhaA enhances the suppressive effects of warming.

Results

Low temperature and humidity increase the survival of bacteria on high-touched dry surfaces in hospital environments

To clarify the relationship between the number of live bacteria on hospital surfaces of different wards and environmental factors (temperature, humidity, and human contact), the data (66 sets) published in our previous manuscript were reused [19] and subjected to principal component analysis (PCA). The data set consisted of the number of

colony-forming units (CFUs) on the high-touched surfaces of different wards [Obstetrics (O), Surgery, (S), or Internal medicine (I)] and environmental factors [temperature (T1–3), humidity (M1–3), and the number of human contacts (N1–3)] in three hospitals of different sizes (designated H, K, and M) located in Sapporo, Japan. PCA revealed a cumulative contribution rate, which was the sum of the first (44.1%) and second (31.4%) principal components (PCs), of 75.5%, and the main composite variables of PC1 and PC2 were humidity (negative impact) and temperature (negative impact), respectively (Fig. 1A). Based on these variables, a scatter plot was created with four groups, consisting of low temperature and high humidity in Group 1, high temperature and high humidity in Group 2, low temperature and low humidity in Group 3, and high temperature and low humidity in Group 4 (Fig. 1A). The groupings were significantly different between the hospitals, which would likely affect the number of viable bacteria on dry surfaces. As expected, the number of live bacteria on the dry surfaces of Group 3, which tended to be low in temperature and humidity, was significantly higher than for the other groups (Fig. 1B and Fig. S1). The ATP level was used as an index for the frequency of human contact, but there was no difference between the groups, indicating that differences in human contact frequency did not affect live-bacterial counts on dry surfaces (Fig. 1C and Fig. S2). A reduction in humidity and temperature promoted the survival of environmental bacteria on high-touched surfaces, and conversely, an increase in temperature to human-skin temperature, as we previously reported [21], decreased the survival of bacteria on dry surfaces, potentially preventing the spread of HAIs.

Warming to human-skin temperature with moderate humidity significantly reduces the survival of human pathogenic bacteria on dry surfaces

We previously found that the number of live bacteria on dry surfaces in hospitals was dramatically influenced by a change in temperature, along with some other contributing environmental factors [19, 20]. We reported preliminary evidence that the survival of pathogenic bacteria was significantly impaired on dry-plastic surfaces warmed to 37°C [21]. In the current study, we employed PCA to investigate the effect of temperature and humidity on the survival of *E. coli* DH5α, and other human pathogenic bacteria, on dry surfaces. First, we determined the survival rate of *E. coli* on a dry plastic surface in an incubator adjusting the humidity from 45%–90% and the temperature from 25°C–37°C, and then estimated the most effective conditions using PCA. The results revealed that the cumulative contribution rate, which was the sum of the first (53.8%) and second (33.3%) PCs, was 87.1%, and the main composite variables of PC1 and PC2 were temperature (negative impact) plus survival rate (positive impact) and humidity (positive impact), respectively. The most significant reduction in the survival rate of *E. coli* was observed at 37°C and 90% humidity, and the least significant effect was observed at 25°C and 45% humidity (Fig. S3). In particular, increasing the temperature to 37°C had a gradual effect on the survival rate, but this effect was considerably diminished at humidity below 60% (Fig. S4), even with warming to human-skin temperature. Furthermore, the warming effect was similarly observed for other human

pathogenic bacteria (*S. aureus*, *P. aeruginosa*, *Acinetobacter baumannii*, and *bla*$_{\text{NDM-5}}$ *E. coli)* as well as *E. coli* DH5α (Fig. 2). However, warming to 37°C had no effect on the spores of *Bacillus subtilis*, but intermittent warming at human-skin temperature significantly reduced the number of live bacteria (Fig. S5). Thus, warming to human-skin temperature along with moderate humidity remarkably suppressed the survival of human pathogenic bacteria on dry surfaces.

Warming of handrail surfaces to human-skin temperature with moderate humidity significantly impairs the survival of human pathogenic bacteria

Finally, we verified the effect of direct heating of a dry handrail surface to human-skin temperature under moderate humidity (55%) on the survival of human pathogenic bacteria. Warming to human-skin temperature was performed using a detachable motorcycle hand heater, and a commercially-available stainless handrail designed for doors was used as an experimental device (Fig. 3A, left). The handrail (at the point closest to the heater) was warmed to human-skin temperature (approximately 37°C), with the temperature gradually decreasing depending on the distance from the heater (Fig. 3A, right). As expected, the survival rate of *E. coli* DH5α decreased significantly on the handrail surface warmed to human-skin temperature (Fig. 3B). The effect became more pronounced with time (Fig. 3C). To confirm this killing effect, we stained live/dead bacteria recovered from each point on the heated handrail. Similar to the CFU data, the relative staining of *E. coli* DH5α significantly decreased with proximity to the

heating zone (Fig. S6). Furthermore, the inhibitory effect of heating was confirmed for other human pathogenic bacteria (*S. aureus*, *P. aeruginosa*, and *A. baumannii)*, but was least effective against *S. aureus* (Fig. 4A). We next identified a dry-resistance factor (NhaA: Na^+/H^+ antiporter) from the Tn-mutant library of *E. coli* DH5α (*n*=243) (Fig. S7), and examined the effect of treatment with an NhaA inhibitor (2-aminoperimidine, 2-AP) (Fig. 4B). As expected, 2-AP significantly enhanced the effect of warming to human-skin temperature on the survival of *S. aureus* on dry surfaces similar to the effect on *A. baumannii*, which is naturally resistant to drying [9] (Fig. 4C). Thus, warming to human-skin temperature under conditions of moderate humidity remarkably impaired the survival rate of human pathogenic bacteria on dry surfaces.

Discussion

Here, we show that environmental temperature and humidity have a great impact on the survival of bacteria on high-touched dry surfaces. In particular, hospital surfaces at low temperature and humidity tended to harbor more live bacteria. Furthermore, we experimentally demonstrated that pathogenic bacteria applied to dry surfaces were quickly killed by warming to human-skin temperature with moderate humidity. Thus, we succeeded in controlling the survival of pathogenic bacteria on dry surfaces simply by warming to human-skin temperature without relying on disinfectants. This has potential implications for preventing the transmission of pathogenic bacteria.

It is recommended by the American Society of Heating, Refrigerating and Air-conditioning Engineers Standard that the temperature in hospitals should be controlled between 20°C–27°C with 40%–60% humidity for comfort [22]. The indoor temperature and humidity varied in the hospitals that we assessed in Japan. For example, the temperature and humidity in hospital “M” were set lower than those in the other hospitals tested, and more live bacteria were detected. Because there was no difference in the amount of ATP (an index of human contact) on the dry surfaces in this hospital, the higher number of live bacteria was not a result of a higher frequency of human contact. Thus, the temperature and humidity arbitrarily set by the air conditioning system affected the microenvironment of the dry surfaces, directly influencing the survival of live bacteria on these surfaces. This confirmed that a low temperature and low humidity are advantageous to the survival of pathogenic bacteria on dry surfaces.

We performed matrix analysis (temperature range: 25°C–37°C, humidity: 45%–90%) on plastic plates to determine the optimal temperature and humidity that could control live pathogenic bacteria on dry surfaces. As expected, in contrast to a low temperature with low humidity, warming to human-skin temperature (37°C) with increasing humidity (~90%) dramatically impaired the survival rates of other pathogenic bacteria as well as *E. coli* DH5α. Two pathogenic bacteria (*S. aureus* and *A. baumannii*), which are known to exhibit resistance to desiccation under dry conditions as a result of a thick cell wall or effective trehalose accumulation [23-25], were also susceptible to heating and humidity, but to a lesser extent than *E. coli*. Heating to human-skin temperature had no effect on

spore-forming bacteria (*B. subtilis*), but intermittent warming (every 2 h) to human-skin temperature significantly reduced (to one-tenth) the number of live bacteria on dry surfaces. This suggested that warming has a potential inhibitory effect on spore-forming *Clostridioides difficile*, which is one of the most common infectious causes of hospital-acquired diarrhea [26, 27]. Thus, warming to human-skin temperature with moderate humidity is likely to have a general suppressive effect on pathogenic bacteria on dry surfaces.

As expected, when a stainless steel handrail device was directly warmed to human-skin temperature using a motorcycle hand heater, the survival rates of pathogenic bacteria significantly decreased. It is reported that bacteria may enter a dormant state stochastically in microbial populations in response to changes in culture conditions such as exposure to antibiotics or heating [28, 29]. However, in our study, the results of live/dead staining confirmed that the pathogenic bacteria had not entered a dormant state and that this did not explain the decrease in cultured cells from the dry surfaces. We used a stainless steel handrail in our experiments, which is a material that is widely available and widely used in public spaces, but it is worth noting that other materials may vary in their ability to conduct heat and therefore the effects of heating may be weakened. In fact, our previous study showed that the survival rate of bacteria differed depending on the material used for the dry surfaces in a hospital environment [30]. Further studies should address this issue by testing materials in terms of the effect of warming to human-skin temperature.

At present, the detailed mechanism by which heating weakens the survival rate of pathogenic bacteria on dry surfaces remains unknown. However, accumulating evidence indicates that temperature affects the survival of pathogenic bacteria on several surfaces by drying. For example, *Salmonella enterica* on an eggshell surface was found to grow under low-temperature (4°C–12°C) with low-humidity (43%) rather than high-temperature (25°C) with high-humidity (85%) [31]. It has also been reported that *S. enterica* or enterohemorrhagic *E. coli* on a plastic tray died more rapidly under hot (25°C) rather than cold conditions (5°C) [32]. Furthermore, it has been reported that pathogens [*E. coli* O157:H7, *Shigella sonnei*, *S. enterica*, *Clostridium perfringens*, hepatitis A virus (HAV), feline calicivirus (FCV), and coliphage PRD1] were killed significantly more effectively under higher humidity conditions [33]. Taken together, these studies support our finding that warming to human-skin temperature can control the survival of pathogenic bacteria on dry surfaces.

We newly identified NhaA as a dry-resistance factor by screening a Tn-insertion mutant library of *E. coli* DH5α. The NhaA transporter is reported to be important for regulating and maintaining the pH in and around bacteria [34], and is extremely well conserved from bacteria to eukaryotes [35]. Although the role of NhaA in drying is not fully understood, it is likely responsible for controlling the leakage of ions from bacterial cells whilst maintaining bacterial structures [36, 37]. A hydrophobic NhaA inhibitor, 2-AP, readily penetrates the bacterial cell wall and binds specifically to three amino

acids of NhaA (binding residues for *E. coli*: N64, F71, H225), inhibiting its transport activity [36, 37]. As expected, a synergistic effect was observed between warming to human-skin temperature and 2-AP for a pathogenic bacterium (*S. aureus*). In the case of staphylococci, which have thick cell walls, it may not be possible to completely disrupt the water balance in bacterial cells by warming to human-skin temperature alone. However, there may be a potential application for 2-AP, which is inexpensive to produce, as a spray for the daily cleaning of dry surfaces in hospital environments.

The control of HAIs confers a significant burden on resources in healthcare settings [38]. Traditional cleaning methods are inefficient for decontamination, and new approaches have been proposed, including disinfectants, steam, automated dispersal systems, and antimicrobial surfaces [11]. Because disinfection using chemical substances raises concerns regarding the emergence of resistance, new treatment strategies are needed [39]. The warming of high-touched dry surfaces may represent a new method of controlling the transmission of pathogenic bacteria in hospitals.

Conclusions

The dry surfaces that our hands come into contact with are crucial mediators of infectious disease transmission via pathogenic bacteria. However, there is currently no reliable method for controlling live pathogenic bacteria on “high-touch” surfaces. In hospitals, frequently-touched surfaces are regularly cleaned with disinfectant, but this

does not prevent nosocomial infections. Here, we found that the survival of pathogenic bacteria can be controlled on dry surfaces simply by warming to human-skin temperature under conditions of moderate humidity. Furthermore, NhaA (Na^+/H^+ antiporter) plays a role in regulating the survival of bacteria on dry surfaces, and the inhibitor 2-AP may have potential applications in controlling pathogenic bacteria on dry surfaces in hospital environments. Our findings may contribute toward creating safer public environments with a decreased risk of infectious disease transmission by employing a novel method that does not rely on chemicals such as disinfectants.

Materials and Methods

Data set reused from our previous study

The data (66 sets) relating to the “number of people,” “temperature,” “humidity,” “CFU numbers,” and “ATP amounts” were reused from our previously published research [19] and subjected to PCA or a multiple comparison test (Table S1). The data sets were collected from three hospitals [at different wards: Internal Medicine (I), Surgery (S), or Obstetrics and Gynecology (O)] of different sizes located in Japan (Sapporo city) from October 2011 to February 2012 [hospital “H” (>500 beds), hospital “K” (100–500 beds), and hospital “M” (<100 beds)].

Bacterial strains used for this study

The following bacteria were used in this study: *E. coli* (DH5α), *P. aeruginosa*

(ATCC27853), *A. baumannii* (ATCC19606), *bla*$_{NDM-5}$ *E. coli* (clinical isolate), *S. aureus* (ATCC29213), and spore-forming *B. subtilis* (laboratory stock). The bacteria were grown in Luria–Bertani (LB) medium (Nacalai Tesque, Kyoto, Japan) or nutrient medium (Nissui Pharma, Tokyo, Japan).

Evaluation of the survival of pathogenic bacteria on dry surfaces under controlled temperature and humidity

To determine the most effective conditions for inhibiting the survival of *E. coli* DH5α on the dry surface of a 96-well plastic plate, various combinations of temperature (25°C–37°C) and humidity (45%–90%) were evaluated using a thermo-hygrostat [IW223 (Yamato Scientific Co., Ltd., Tokyo, Japan) or KCL-2000W (EYELA, Tokyo Rikakikai Co., Ltd., Tokyo, Japan)]. In brief, the bacteria were suspended in LB liquid medium to 1.0×10^{10} CFU/mL, and 5 µL of solution was spotted onto a 96-well plate (U-type). After drying completely (T0), the plate was incubated in the thermo-hygrostat for 18 h (T18). The dried bacteria in the wells were suspended in 100 µL of LB liquid medium and the number of live bacteria was counted. The live bacterial count was expressed as the ratio of the decreased value at “T18” to the value at “T0.” The data sets for “temperature,” “humidity,” and “the survival rate of *E. coli*” were used for PCA or a multiple comparison test (Table S2). Other pathogenic bacteria (*P. aeruginosa* ATCC27853, *A. baumannii* ATCC19606, *bla*$_{NDM-5}$ *E. coli*, and *S. aureus* ATCC29213) were similarly verified under the most and least effective conditions for the survival of *E. coli* DH5α on the dry surface. In addition, the effect of intermittent warming to

human-skin temperature on spore-forming bacteria (*B. subtilis*) was verified (see the protocol in Fig. S5A).

Evaluation of the survival of human pathogenic bacteria on handrail surfaces warmed to human-skin temperature with moderate humidity

Commercially available stainless steel (length: 34 cm, diameter: 3.3 cm) placed sideways on wooden boards was used to simulate a “handrail dry surface” with a 9.2 cm-long-grip heater (OPMID, Osaka, Japan) attached to one end of the steel. This handrail device was placed into the thermo-hygrostat adjusted to 15°C (temperature) and 55% (humidity). The handrail temperature was monitored by thermography (FLIR ONE Pro, Teledyne Flir, Wilsonville, OR, USA). The output of the heater had three settings (5 W, 7.5 W, 10 W) and the 10 W setting was selected as this heated the adjacent handrail to approximately 37°C. The number of live bacteria on the handrail was determined as follows. In brief, the bacteria were suspended in 0.85% saline to a concentration of 1.5×10^{10} CFU/mL. Each 10 μL of solution was dropped onto a release paper of double-sided tape cut into 1.5 cm squares (1.5×10^{8} CFU/spot). In some experiments, 2-AP (~100 μM) was added to the solution. After drying, the bacteria-spotted tapes were applied at different distances [0 cm (right next to the heater) – 15 cm] from the heater on the handrail device, and then incubated in the thermo-hygrostat adjusted at 15°C (temperature) and 55% (humidity) for ~72 h (T18, T48, T72). Each tape was then collected, suspended in 500 μL of PBS, and the number of live bacteria was counted. The live bacterial count was expressed as the ratio of the

decreased value at “T18–T72” to the value at “T0.” The viability of *E. coli* on the handrail was also quantified by an image analyzer (Kyence, Osaka, Japan) after Live/Dead BacLight staining (Molecular Probes, Eugene, OR, USA).

Screening of a mutant with the least dry resistance from a Tn-insertion *E. coli* DH5α library and the generation of strains in which the gene was disrupted/reintroduced

A Tn-insertion mutant library of *E. coli* DH5α was constructed using the EZ-Tn5™ <KAN-2>Tnp Transposome™ kit (AR Brown Co., Ltd., Tokyo, Japan), according to the manufacturer's protocol. The library consisted of 243 mutant strains. The bacterial solution (5 μL, approximately 1.0×10^5 CFU/spot) was spotted onto a 96-well plastic plate (U type), and then dried at room temperature. The number of CFUs was compared between the immediately dried (T0) and 24-h incubated (T24) samples at room temperature (approximately 22°C), and the results were expressed as a ratio (T24/T0). The Tn-inserted gene of a mutant strain with the least dry resistance was identified by arbitrary primed PCR, according to a previous report [40]. The primers used for PCR were as follows: 1st PCR TnF: 5′-GCA ATG TAA CAT CAG AGA GAG ATT TTG AG-3′, 1st PCR R: 5′-GGC CAC GCG TCG ACT AGT ACN NNN NNN NNN GAT AT-3′, 2nd PCR TnF: 5′-AGC TTC AGG GTT GAG ATG TG-3′, 2nd PCR R: 5′-GGC CAC GCG TCG ACT AGT AC-3′. Amplified DNA was sequenced, and then genes were determined based on matches to the NCBI database (https://www.ncbi.nlm.nih.gov/). Because *nhaA* was the only site of Tn-insertion in the

mutant strain with markedly reduced dry resistance, strains in which the gene was disrupted by recombination and reintroduced by complementation were established. In brief, recombination was performed using a kanamycin resistance cassette inserted into sites 50 bp upstream (*nhaA*-F 5′-GCG GGG TAA AAT AGT AAA AAC GAT CTA TTC ACC TGA AAG AGA AAT AAA AAA ATA ATT A-3′) and downstream (*nhaA*-R 5′-TGA TAA CAA TGA AAA GGG AGC CGT TTA TGG CTC CCC GGT AAA CCG TCC TGT AAT ACG AC-3′) of the *nhaA* gene in the cloning site of plasmid pKD46 encoding the recombinase. The complementary strain was established by transformation of pSTV28 (Clontech Takara Cellartis, Shiga, Japan) containing the *nhaA* gene.

Three-dimensional (3D) structure of NhaA and the binding sites of the 2-aminoperimidine inhibitor

The 3D structures of annotated protein sequences of *E. coli* NhaA (MMDB ID: 33841, PDB ID: 1ZCD) were acquired from the NCBI structure library (https://www.ncbi.nlm.nih.gov/), and the chemical structure of the specific inhibitor (2-aminoperimidine: PubChem ID: 112416) was obtained from the PubChem compound library at the same site.

PCA and statistical analysis

The two datasets [field study (temperature, humidity, and the number of people in three city hospitals in Sapporo) (See Table S1), experimental study (temperature, humidity, and the survival rate of *E. coli*) (See Table S2)] were separately compressed into

two-component synthetic variables by PCA using R (Version 1.0.136), and the groups were visualized in Excel (Version 14.7.3). Multiple comparison tests were performed using the Bonferroni/Dunn method or the Steel–Dwass method. A *p*-value of less than 0.05 ($p<0.05$) was considered significant.

References

1. Facciolà A, Pellicanò GF, Visalli G, Paolucci IA, Venanzi Rullo E, Ceccarelli M, D'Aleo F, Di Pietro A, Squeri R, Nunnari G, La Fauci V. The role of the hospital environment in the healthcare-associated infections: a general review of the literature. Eur Rev Med Pharmacol Sci 2019; 23:1266-1278.

2. Peters A, Schmid MN, Parneix P, Lebowitz D, de Kraker M, Sauser J, Zingg W, Pittet D. Impact of environmental hygiene interventions on healthcare-associated infections and patient colonization: a systematic review. Antimicrob Resist Infect Control 2022;11:38.

3. Marcel JP, Alfa M, Baquero F, Etienne J, Goossens H, Harbarth S, Hryniewicz W, Jarvis W, Kaku M, Leclercq R, Levy S, Mazel D, Nercelles P, Perl T, Pittet D, Vandenbroucke-Grauls C, Woodford N, Jarlier V. Healthcare-associated infections: think globally, act locally. Clin Microbiol Infect 2008;14:895-907.

4. Al-Tawfiq JA, Tambyah PA. Healthcare associated infections (HAI) perspectives. J Infect Public Health 2014;7:339-344.

5. Zimlichman E, Henderson D, Tamir O, Franz C, Song P, Yamin CK, Keohane C, Denham CR, Bates DW. Health care-associated infections: a meta-analysis of costs and financial impact on the US health care system. JAMA Intern Med 2013;173:2039-2046.

6. Drohan SE, Levin SA, Grenfell BT, Laxminarayan R. Incentivizing hospital infection control. Proc Natl Acad Sci USA 2019;116:6221-6225.

7. Kramer A, Schwebke I, Kampf G. How long do nosocomial pathogens persist on

inanimate surfaces? A systematic review. BMC Infect Dis 2006;6:130.

8. Sahin S, Mogulkoc MN, Kürekci C. Disinfectant and heavy metal resistance profiles in extended spectrum β-lactamase (ESBL) producing *Escherichia coli* isolates from chicken meat samples. Int J Food Microbiol 2022;377:109831.

9. Bolla JR, Howes AC, Fiorentino F, Robinson CV. Assembly and regulation of the chlorhexidine-specific efflux pump AceI. Proc Natl Acad Sci USA 2020;117:17011-17018.

10. El-Banna T, Abd El-Aziz A, Sonbol F, El-Ekhnawy E. Adaptation of *Pseudomonas aeruginosa* clinical isolates to benzalkonium chloride retards its growth and enhances biofilm production. Mol Biol Rep 2019;46:3437-3443.

11. Dancer SJ. Controlling hospital-acquired infection: focus on the role of the environment and new technologies for decontamination. Clin Microbiol Rev 2014;27:665-690.

12. Williamson DA, Carter GP, Howden BP. Current and Emerging Topical Antibacterials and Antiseptics: Agents, Action, and Resistance Patterns. Clin Microbiol Rev 2017;30:827-860.

13. Protano C, Cammalleri V, Romano Spica V, Valeriani F, Vitali M. Hospital environment as a reservoir for cross transmission: cleaning and disinfection procedures. Ann Ig 2019;31:436-448.

14. Yang L, Liu Y, Wu H, Song Z, Høiby N, Molin S, Givskov M. Combating biofilms. FEMS Immunol Med Microbiol 2012;65:146-157.

15. Stone W, Tolmay J, Tucker K, Wolfaardt GM. Disinfectant, Soap or Probiotic

Cleaning? Surface Microbiome Diversity and Biofilm Competitive Exclusion. Microorganisms. 2020;8:1726.

16. Lebre PH, De Maayer P, Cowan DA. Xerotolerant bacteria: surviving through a dry spell. Nat Rev Microbiol 2017;15:285-296.

17. Alpert P. Constraints of tolerance: why are desiccation-tolerant organisms so small or rare? J Exp Biol 2006;209:1575-1584.

18. Okubo T, Osaki T, Nozaki E, Uemura A, Sakai K, Matushita M, Matsuo J, Nakamura S, Kamiya S, Yamaguchi H. Walker occupancy has an impact on changing airborne bacterial communities in an underground pedestrian space, as small-dust particles increased with raising both temperature and humidity. PLoS One. 2017;12:e0184980.

19. Watanabe R, Shimoda T, Yano R, Hayashi Y, Nakamura S, Matsuo J, Yamaguchi H. Visualization of hospital cleanliness in three Japanese hospitals with a tendency toward long-term care. BMC Res Notes 2014;7:121.

20. Yano R, Shimoda T, Watanabe R, Kuroki Y, Okubo T, Nakamura S, Matsuo J, Yoshimura S, Yamaguchi H. Diversity changes of microbial communities into hospital surface environments. J Infect Chemother 2017;23:439-445.

21. Shimoda T, Okubo T, Enoeda Y, Yano R, Nakamura S, Thapa J, Yamaguchi H. Effect of thermal control of dry fomites on regulating the survival of human pathogenic bacteria responsible for nosocomial infections. PLoS One 2019;14:e0226952.

22. ASHRAE Standard 90.1–2022: Energy standard for sites and buildings except

low-rise residential buildings. American Society of Heating, Refrigerating and Air-conditioning Engineers, Atlanta, GA.

23. Almatroudi A, Tahir S, Hu H, Chowdhury D, Gosbell IB, Jensen SO, Whiteley GS, Deva AK, Glasbey T, Vickery K. *Staphylococcus aureus* dry-surface biofilms are more resistant to heat treatment than traditional hydrated biofilms. J Hosp Infect 2018;98:161-167.
24. Wendt C, Dietze B, Dietz E, Rüden H. Survival of *Acinetobacter baumannii* on dry surfaces. J Clin Microbiol 1997;35:1394-1397.
25. Zeidler S, Müller V. The role of compatible solutes in desiccation resistance of *Acinetobacter baumannii*. Microbiologyopen 2019;8:e00740.
26. Czepiel J, Dróżdż M, Pituch H, Kuijper EJ, Perucki W, Mielimonka A, Goldman S, Wultańska D, Garlicki A, Biesiada G. *Clostridium difficile* infection: review. Eur J Clin Microbiol Infect Dis 2019;38:1211-1221.
27. Guh AY, Kutty PK. *Clostridioides difficile* Infection. Ann Intern Med 2018;169:ITC49-ITC64.
28. Lewis K. Persister cells. Annu Rev Microbiol 2010;64:357-372.
29. Zhang B, Fu Y, Wang F, Yang J, Pan Z, Huang M, Shen K, Shen C. Multidrug-resistant enteroaggregative *Escherichia coli* (EAEC) enters dormant state during heat treatment: A potential hazard in municipal sludge. Environ Pollut 2022;305:119312.
30. Shimoda T, Yano R, Nakamura S, Yoshida M, Matsuo J, Yoshimura S, Yamaguchi H. ATP bioluminescence values are significantly different depending upon material

surface properties of the sampling location in hospitals. BMC Res Notes 2015;8:807.

31. Kingsbury JM, Thom K, Soboleva T. Effect of Storage Temperature on the Survival of New Zealand Egg-Associated *Salmonella* Isolates in and on Eggs. J Food Prot 2019;82:2161-2168.

32. Hokunan H, Koyama K, Hasegawa M, Kawamura S, Koseki S. Survival Kinetics of *Salmonella enterica* and Enterohemorrhagic *Escherichia coli* on a Plastic Surface at Low Relative Humidity and on Low-Water Activity Foods. J Food Prot 2016;79:1680-1692.

33. Stine SW, Song I, Choi CY, Gerba CP. Effect of relative humidity on preharvest survival of bacterial and viral pathogens on the surface of cantaloupe, lettuce, and bell peppers. J Food Prot 2005;68:1352-1358.

34. Padan E, Tzubery T, Herz K, Kozachkov L, Rimon A, Galili L. NhaA of Escherichia coli, as a model of a pH-regulated Na^+/H^+ antiporter. Biochim Biophys Acta 2004;1658:2-13.

35. Xiang M, Feng M, Muend S, Rao R. A human Na^+/H^+ antiporter sharing evolutionary origins with bacterial NhaA may be a candidate gene for essential hypertension. Proc Natl Acad Sci USA 2007;104:18677-18681.

36. Padan E, Gerchman Y, Rimon A, Rothman A, Dover N, Carmel-Harel O. The molecular mechanism of regulation of the NhaA Na^+/H^+ antiporter of *Escherichia coli*, a key transporter in the adaptation to Na^+ and H^+. Novartis Found Symp 1999;221:183-96.

37. Padan E, Venturi M, Gerchman Y, Dover N. $Na^{(+)}/H^{(+)}$ antiporters. Biochim Biophys

Acta 2001;1505:144-157.
38. Drohan SE, Levin SA, Grenfell BT, Laxminarayan R. Incentivizing hospital infection control. Proc Natl Acad Sci USA 2019;116:6221-6225.
39. Rutala WA, Barbee SL, Aguiar NC, Sobsey MD, Weber DJ. Antimicrobial activity of home disinfectants and natural products against potential human pathogens. Infect Control Hosp Epidemiol 2000;21:33-38.
40. Saavedra JT, Schwartzman JA, Gilmore MS. Mapping Transposon Insertions in bacterial genomes by arbitrarily primed PCR. Curr Protoc Mol Biol 2017;118:15.

Acknowledgments

We thank Mr Kazuki Morinaga and Ms Nao Fujii for their help with the laboratory research. A professional proofreading company, Edanz (https://jp.edanz.com/ac), edited a draft of this manuscript.

Supplementary Information

Table S1: Data sets (temperature, humidity, number of people, and the number of bacteria (CFU) / the frequency of human contact (ATP) in three city hospitals in Sapporo) reused from our previously published manuscript [19]. "CFU" indicates the number of live bacteria on dry surfaces in hospitals, "ATP" shows the human-contact frequency for these surfaces.

Table S2: Data sets obtained from experimental matrix analysis (temperature, humidity, and the survival rate of *E. coli*). "Survival rate" indicates the value (number of live *E. coli*) for "T18" vs "T0."

Fig. S1: Comparison of the number of live bacteria at each sampling site among the four groups classified by environmental factors. Group 1 was low temperature and high humidity [total 108: n=12 (each bar)], group 2 was high temperature and high humidity [total 135: n=15 (each bar)], group 3 was low temperature and low humidity [total 144: n=16 (each bar)], and group 4 was high temperature and low humidity [total 21: n=21 (each bar)]. See the detailed sampling sites in Table S1. Bars show the average ± SD. *, $p<0.05$ with statistical significance between specific groups.

Fig. S2: Comparison of the amount of ATP at each sampling site among the four groups classified by environmental factors. Group 1 was low temperature and high humidity, [total 117: n=12 (each bar)], group 2 was high temperature and high humidity [total

135: *n*=15 (each bar)], group 3 was low temperature and low humidity [total 152: *n*=17 (each bar excluding "locker, *n*=16")], and group 4 was high temperature and low humidity [total 189: *n*=21 (each bar)]. See the detailed sampling sites in Table S1. Bars show the average ± SD. *, $p<0.05$ with statistical significance between specific groups.

Fig. S3: Warming to human-skin temperature with moderate humidity significantly reduces the survival rate of human pathogenic bacteria on dry surfaces. **A.** PCA scatter plot showing the impact on the survival rate of *E. coil* of temperature (25°C–37°C) and humidity (45%–90%). PC1-factor loading shows -0.646 (T: temperature), -0.288 (M: humidity), and 0.707 (S: the survival rate of *E. coli*). PC2-factor loading shows -0.408 (T) and 0.913 (M). Six runs were performed for each combination of matrices. **B.** Comparison of the survival rate of *E. coli* on dry surfaces (18 h/0 h) with 45%–90% humidity at 25°C. Bars (*n*=6) show the average ± SD. *, $p<0.05$, with a statistically significant difference compared with the conditions for highest survival (T25/M45). **C.** Comparison of the survival rate of *E. coli* on dry surfaces (18 h/0 h) with 45%–90% humidity at 37°C. Bars (*n*=6) show the average ± SD. *, $p<0.05$, with a statistically significant difference compared with the conditions for highest survival (T37/M45).

Fig. S4: Detailed comparison of the survival rates of *E. coli* on dry surfaces (18 h/0 h) at 25°C–37°C and 45%–90% humidity using the data obtained from Fig. S3. Bars (*n*=6) show the average ± SD. *, $p<0.05$, with a statistically significant difference compared with the conditions for highest survival for each panel.

Fig. S5: Intermittent warming to human-skin temperature significantly reduced the number of spore-forming *B. subtilis*. **A.** Graphs show the schedule of intermittent warming and the temperature (left) and humidity (right) [blue: -B- (repeating 22°C with 45% humidity and 37°C with 90% humidity with an interval of 2 h)]. Fixed schedules were included as controls [red: -A- (22°C and 45%), green: -C- (37°C and 90%)]. **B.** Temperature and humidity monitoring with a data logger (AD-6324SET, AD Discover Precision, Tokyo, Japan). Red, temperature. Blue, humidity. Arrows indicate the timing of removal of the plate from the incubator. **C.** Effect of intermittent warming at human-skin temperature on decreasing the number of *B. subtilis* compared with *E. coli*. **, $p<0.01$, with a statistically significant difference compared with those of “red bar A.”

Fig. S6: Validity of the results that heating of handrails to human-skin temperature kills *E. coli* DH5α by live/dead staining. Images show representative staining patterns (× 1,000). Area (red or green) was measured as the number of pixels by ImageJ software. Survival rate is shown as a ratio (T24/T0). Experiments were performed at least three times. Bars show the average ± SD. *, $p<0.05$, with a statistically significant difference compared with “cont.”

Fig. S7: Survival rate of Tn-inserted *E. coli* mutants [library with 243 mutants (red numbers)] on dry surfaces, and confirmation of its efficacy using *nhaA*-disrupted and

-reintroduced strains. **A.** The number of CFUs was compared between immediately-dried (T0) and 24-h incubated (T24) samples, and the results were expressed as a ratio (T24/T0). Red arrow indicates the mutant (no. 75) with the lowest resistance to desiccation among the library. **B.** Loss of dry resistance in the *nhaA*-disrupted strain and recovery in the reintroduced strain. The experiment was performed at least three times. Bars show the average ± SD. *, $p<0.05$, with a statistically significant difference compared with "*E. coli* DH5α" for each day. $, $p<0.05$, with a statistically significant difference compared with "*E. coli* DH5αΔ*nhaA*::*nhaA*" for each day.

Figure Legends

Fig. 1. Low temperature and humidity in hospitals increases the survival rate of bacteria on high-touched dry surfaces

A. PCA scatter plot showing four groups (Groups 1–4) with distinct environmental conditions [temperature (T: T1–3), humidity (M: M1–3), and number of people (N: N1–3)] in three hospitals [designated H, K, and M]. PC1-factor loading shows 0.248(T1)/ 0.275(T2)/ 0.297(T3), -0.419(M1)/ -0.398(M2)/ -0.424(M3), and -0.306(N1)/ -0.265(N2)/ -0.312(N3) (N: number of people at that time). PC2-factor loading shows -0.425(T1)/ -0.415(T2)/ -0.383(T3) and -0.404(N1)/ -0.392(N2)/ -0.423(N3). As specified in the text, the data (66 sets) published in our previous manuscript were reused [19]. "O" ward, Obstetrics. "S" ward, Surgery. "I" ward, Internal medicine. "%," cumulative contribution rates for PC1 and PC2. See Table S1. **B.** Comparison of the number of live bacteria (CFU) on dry surfaces between Groups 1–4. Bars [Group 1 (n=108), Group 2 (n=135), Group 3 (n=144), and Group 4 (n=189)] show the average ± SD. *, p<0.05, with a statistically significant difference between the combinations connected by a line. **C.** Comparison of the amount of ATP (as an index for the frequency of "human contact") on dry surfaces between Groups 1–4. Bars [Group 1 (n=117), Group 2 (n=135), Group 3 (n=157), and Group 4 (n=189)] show the average ± SD.

Fig. 2. The warming of dry surfaces to human-skin temperature affects the survival of human pathogenic bacteria [*S. aureus*, *P. aeruginosa*, *A. baumannii*, and *bla*$_{NDM\text{-}5}$ *E. coli* (NDM-5)] as well as *E. coli* DH5α

A. Survival rate of the bacteria under the most effective conditions (T 37°C/M 90%). "Survival rate" shows the ratio of CFUs at "18 h" / "0 h" after drying. Experiments were performed at least three times. Bars show the average ± SD. *, p<0.05, with a statistically significant difference compared with immediately after drying ("0 h"). **B.** Survival rate of the bacteria under the least effective conditions (T 15°C/M 55%). See above. **C.** Survival rates (%) of the bacteria between the most effective [T 37°C/M 90% (A)] and the least effective [T 15°C/M 55% (B)] conditions.

Fig. 3. Heating of handrail surface to human-skin temperature with moderate humidity significantly impairs the survival of *E. coli* DH5α

A. Handrail device and heatmap showing the temperature distribution across the handrail device when connected to the heater. Warming was performed to conditions of 15°C and 55% humidity (T 15°C/M 55%). Red star shows the location of the heater on the handrail. **B.** Survival rate of *E. coli* DH5α at each measurement location. The experiment was performed at least three times. Values show the ratio of CFUs between immediately after drying "0 h" and "18 h" after drying. Images show representative colonies of smear samples on LB agar plates collected from the handrail. Dashed and solid lines show the changing temperature due to heating. Bars show the average ± SD. *, p<0.05, with a statistically significant difference compared with the value immediately after drying "0 h." $, p<0.05, with a statistically significant difference compared with the "Control (without heating)" value. **C.** Effect of handrail warming to human-skin temperature on the survival of *E. coli* DH5α over 72 h. See above. Bar

shows the average ± SD. *, $p<0.05$, with a statistically significant difference compared with the value immediately after drying “0 h” for each time course. $, $p<0.05$, with a statistically significant difference compared with the “Control (without heating)” value for each time course.

Fig. 4. Heating of the handrail surface to human-skin temperature with moderate humidity significantly impairs the survival of other pathogenic bacteria (*S. aureus*, *P. aeruginosa*, *A. baumannii*) as well as *E. coli* DH5α, and an inhibitor (2-AP) of NhaA, which regulates dry resistance, enhances the warming effect against some bacteria

A. Survival rates of human pathogens (*S. aureus*, *P. aeruginosa*, *A. baumannii*) and *E. coli* DH5α on the handrail device warmed to human-skin temperature. Warming was performed under conditions of 15°C and 55% humidity (T 15°C/M 55%). Values show the ratio of CFUs between immediately after drying “0 h” and “18 h” after drying. Bars show the average ± SD. *, $p<0.05$, with a statistically significant difference compared with the value immediately after drying “0 h.” $, $p<0.05$, with a statistically significant difference compared with the “Control (without heating)” value. **B**. 3D structures of annotated protein sequences of *E. coli* NhaA (MMDB ID: 33841 PDB ID: 1ZCD) acquired from the NCBI Structure library (https://www.ncbi.nlm.nih.gov/), and the chemical structure of the specific inhibitor (2-aminoperimidine, 2-AP: PubChem ID: 112416) obtained from the PubChem Compound library at the same site. Yellow marks indicate binding sites for 2-AP. Among the pathogens used in the experiment, only *S.*

aureus replaced “N64” with “A64.” Red ladder, extracellular membrane. Blue ladder, inner membrane. **C.** 2-AP enhances the warming effect on some bacteria (*S. aureus* and *A. baumannii*). See above. Bars show the average ± SD. \$, $p<0.05$, with a statistically significant difference when compared with the value immediately after drying “0 h.” *, $p<0.05$, with a statistically significant difference when compared with those without 2-AP at “18 h after drying.”

Figure1

Click here to access/download;Figure;Fig1.tiff

Fig. 1

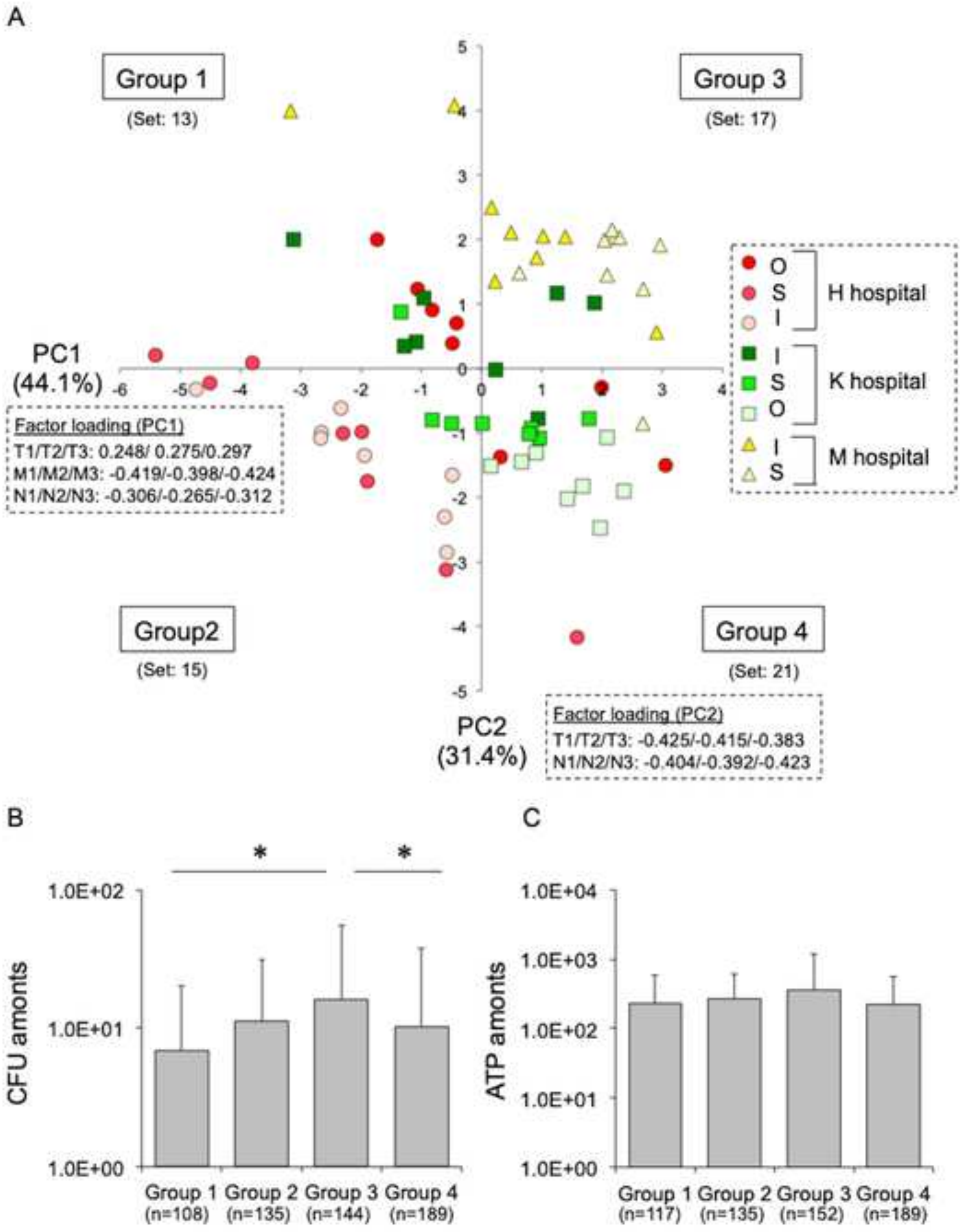

Figure2 Click here to access/download;Figure;Fig2.tiff

Fig. 2

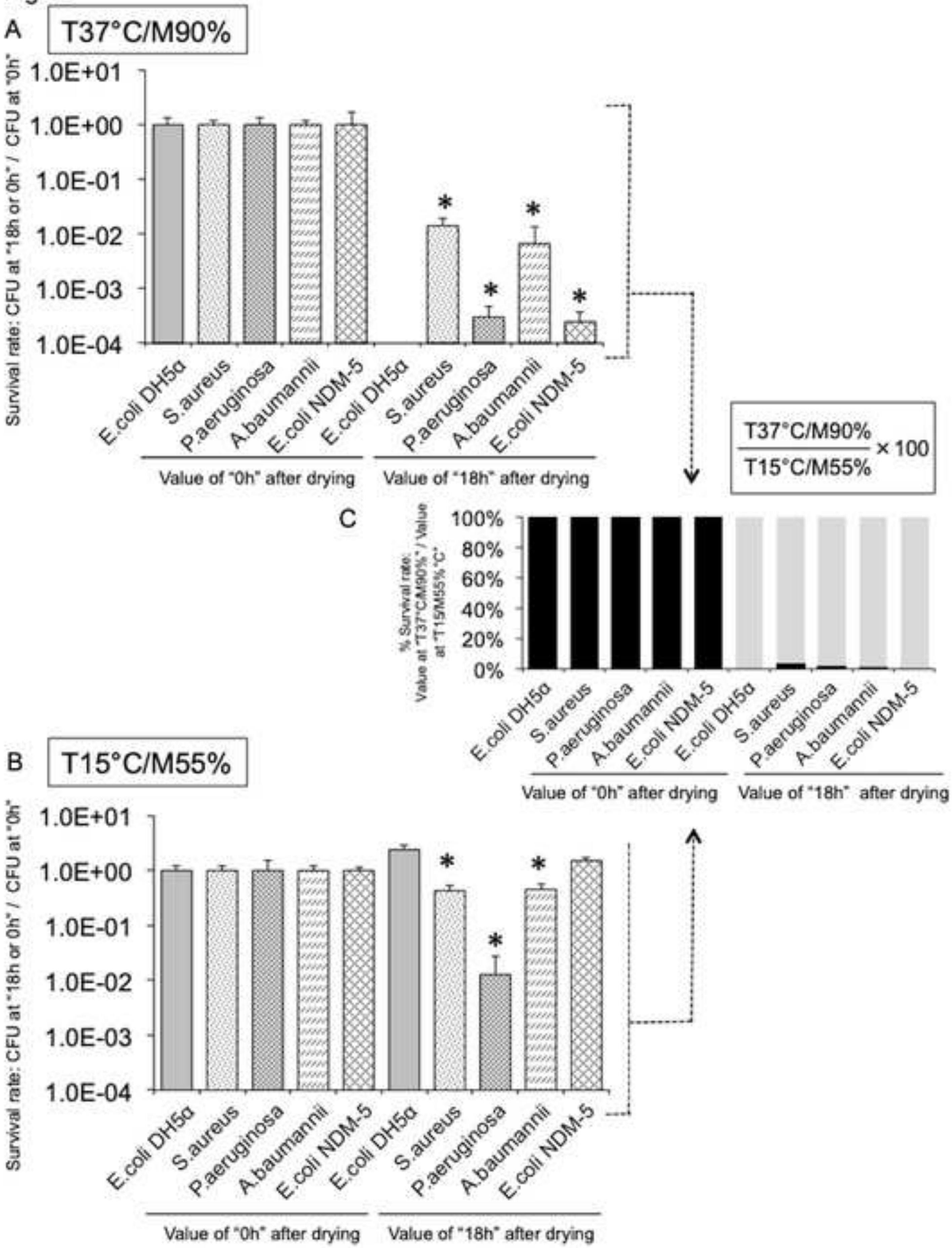

Figure3 Click here to access/download;Figure;Fig3.tiff

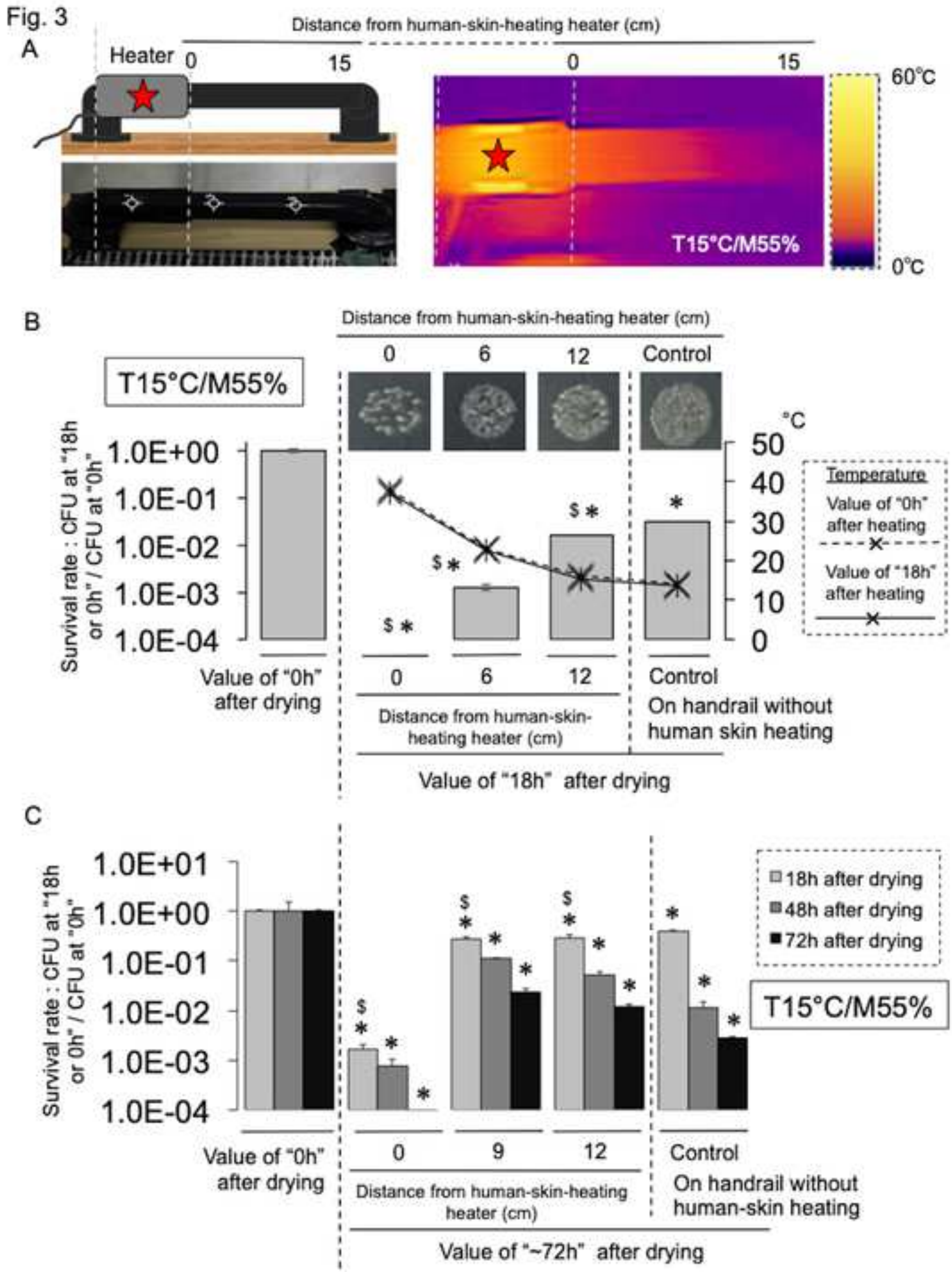

Figure4

Click here to access/download;Figure;Fig4.tiff

Fig. 4

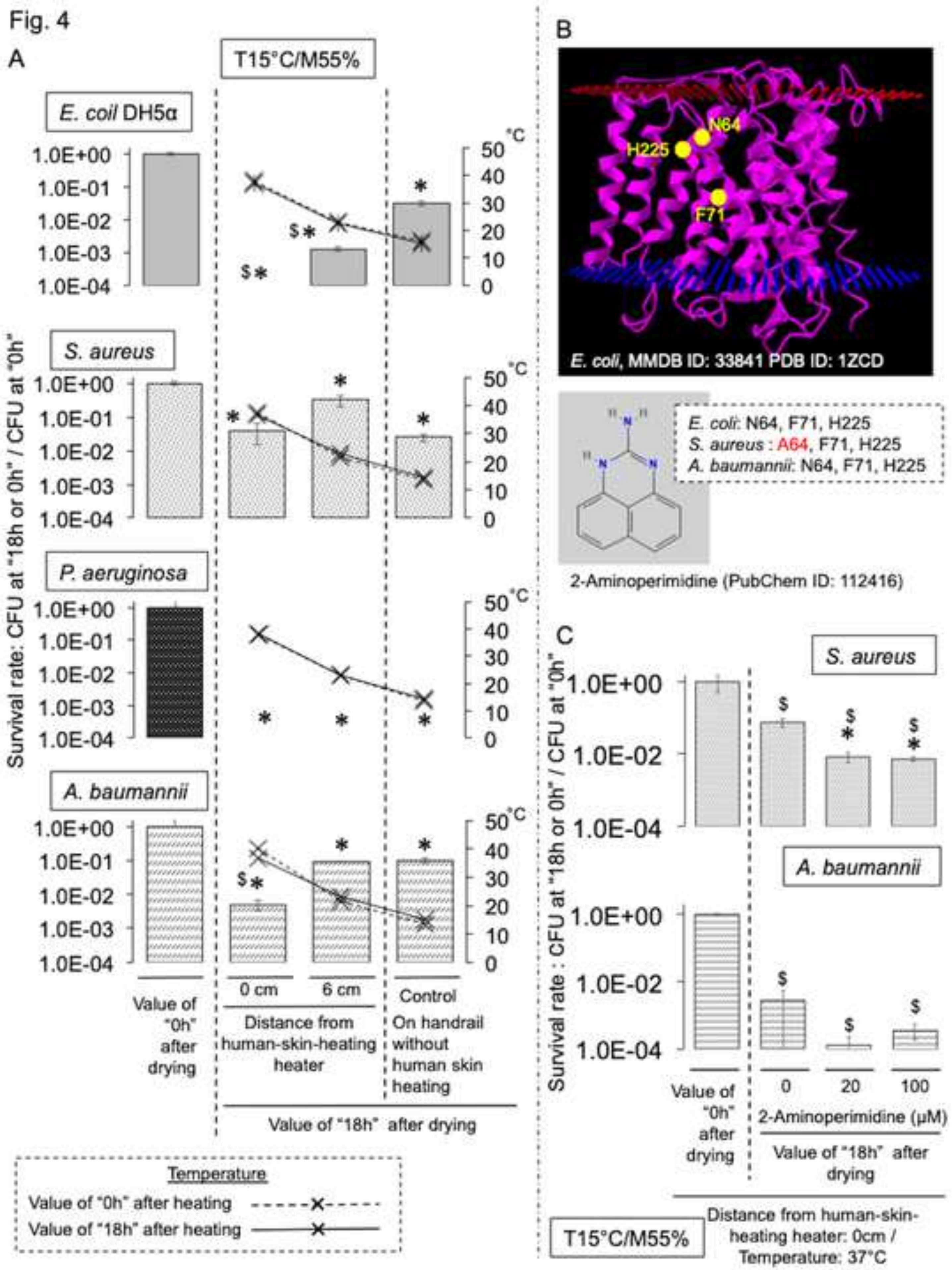

Click here to access/download
Supporting Information
Table S1 20230323.pdf

Table S2

Click here to access/download
Supporting Information
Table S2 20230323.pdf

Fig S1

Click here to access/download
Supporting Information
FigS1 20230323.pdf

Fig S2

Click here to access/download
Supporting Information
FigS2 20230323.pdf

Fig S3

Click here to access/download
Supporting Information
FigS3 20230323.pdf

Fig S4

Click here to access/download
Supporting Information
FigS4 20230323.pdf

Fig S5

Click here to access/download
Supporting Information
FigS5 20230323.pdf

Fig S6

Click here to access/download
Supporting Information
FigS6 20230323.pdf

Fig S7

Click here to access/download
Supporting Information
FigS7 20230323.pdf
