# Supplementary material for: Human pathogenic bacteria on high-touch dry surfaces can be controlled by warming to human-skin temperature under moderate humidity
Source: PLoS One. 2023 Sep 20;18(9):e0291765. doi: 10.1371/journal.pone.0291765 (PMC10511134; doi:10.1371/journal.pone.0291765)
Supplement: Attachment — Submitted filename: The manuscript PONE.docx [file pone.0291765.s011.docx]

Dear Editor

After reviewing the manuscript PONE-D-23-08650 titled " Human pathogenic bacteria on high-touched dry surfaces can be controlled by warming to human-skin temperature under moderate humidity, I found it very well written and clearly presented the data. I found it acceptable for publication, however, there are some points that I think they should be cleared. I have already highlighted them on the manuscript and added a sticky note stating my point. In addition, I found the quality of the figures is not of a publishable grade, especially the text on them, they should be reproduced in a better quality.
