# Supplementary material for: Human pathogenic bacteria on high-touch dry surfaces can be controlled by warming to human-skin temperature under moderate humidity
Source: PLoS One. 2023 Sep 20;18(9):e0291765. doi: 10.1371/journal.pone.0291765 (PMC10511134; doi:10.1371/journal.pone.0291765)
Supplement: Attachment — Submitted filename: Response to Reviewers 20230823.doc [file pone.0291765.s012.doc]

**REPLIES TO REVIEWER’S COMMENTS**

Ref. ID: PONE-D-23-08650

**Human pathogenic bacteria on high-touched dry surfaces can be controlled by warming to human-skin temperature under moderate humidity**

**To Reviewer 1:**

**Comment 1:**

Excellent work, data clearly show the effect of temp and humidity on survival bacteria on high touch surfaces; in addition, the authors attempted to look into the underlying mechanism as well. Even though "high touched" surfaces were defined in the manuscript, "high touch" surfaces would be preferable.

**Response:** According to the comment, we corrected it throughout the text, including the title.

**To Reviewer 2:**

**Comment 1:**

After reviewing the manuscript PONE-D-23-08650 titled " Human pathogenic bacteria on high-touched dry surfaces can be controlled by warming to human-skin temperature under moderate humidity, I found it very well written and clearly presented the data. I found it acceptable for publication, however, there are some points that I think they should be cleared. I have already highlighted them on the manuscript and added a sticky note stating my point. In addition, I found the quality of the figures is not of a publishable grade, especially the text on them, they should be reproduced in a better quality.

**Response:** According to the comments, all corrections have been made to the points written in the text (See below), as well as improved the quality of the figures (Tiff files) with publishable grade. Specifically, the image resolution has been increased to 2,000 pixels per inch.

**Responses to Highlighted points on the manuscript**

**Comment on line 50:**

Why ineffective? some disinfectants are certainly effective.

**Response:** It didn't mean that it was ineffective, but that disinfection measures alone were not enough. We apologize for the lack of explanation. According to the comment, we corrected it.

**Comment on line 196:**

90% humidity is not a moderate as you said that less than 60% there was no effect for the humidity.

**Response:** According to the comment, we corrected it.

**Comment on line 220:**

Does the S. aureus also have this dry resistance factor as you found that in the E. coli library ?

**Response:** Yes, *S. aureus* is also known to show dry resistant. According to the comment, we have quoted the references in the revised text.

**Comment on line 227:**

In real life you might be able to control temperature but humidity is not that easy to control especially at high percentages. In general, we cant increase the humidity of hospital environments beyond a specific percentage as this might lead to other negative conditions that might surpass the benefits of decreasing the bacterial numbers on high touch surfaces. for example, might lead to the growth of mold in certain damp areas and might increase survival of desiccation resistant bacteria.

**Response:** We agree with the point that the reviewer pointed out. According to the comment, we added the explanation that high humidity has negative effects such as mold growth in the revised text.

**Comment on line 261:**

???? is not this Clostridium.

**Response:** Very sorry. As pointed out, it may not be correct to describe this bacterium as a primary pathogen of diarrhea. As pointed out by the reviewer, the text has been revised to state that diarrhea due to this bacterial infection is increasing in healthcare facilities. We hope you are satisfied with this correction.

**Comment on line 312:**

This is not a new method of cleaning.

**Response:** According to the comment, we corrected it properly.

**Comment on line 316:**

Bacteria may also develop resistance to this slight increase in the temperature. Some bacteria develop resistance to dessication by acquiring genes from other bacteria.

**Response:** As pointed out by the reviewer, we can't deny the possibility that, like drug-resistant bacteria, they will become resistant to human-skin warming. According to the comment, we added a note about that.

**Comment on line 629:**

Evidenced by live/dead staining.

**Response:** According to the comment, we corrected it.

**To Reviewer 3:**

**Comment 1:**

The manuscript entitled “Human pathogenic bacteria on high-touched dry surfaces can be controlled by warming to human-skin temperature under moderate humidity” investigated the dynamics of bacteria on dry surfaces in hospitals and showed experimentally that human pathogenic bacteria on dry surfaces (handrails) can be controlled by warming of the surfaces to human-skin temperature under moderate humidity. Furthermore, they demonstrated that NhaA, an Na+ /H+ antiporter, is a crucial factor in supporting the survival of bacteria under dry conditions, and that an inhibitor 2-AP of NhaA enhances the suppressive effects of warming. It contains some very interesting results, and it is suitable for publication. In their experiment, they used a commercially available stainless steel handrail designed for doors as an experimental device. I would like to know if they have tested or intend to test with another type of material different from the one used in order to check if the result will be similar.

**Response:** Thank you for the comment. The materials used in experiments on the effect of warming to human-skin temperature under control of humidity have not yet been thoroughly verified except for polyethylene, which is the material of the well plate, and stainless steel, which is used for handrails. Meanwhile, other materials will be tested in the future. Specifically, in collaboration with handrail manufacturers, we plan to use materials that have already been installed in public environments that people touch frequently and verify them on devices capable of more accurate temperature control. Meanwhile, although it has already been published (BMC Res Notes. 2015 Dec 21;8:807. doi: 10.1186/s13104-015-1757-9), we have found that the number of surviving bacteria on high-frequency contact surfaces varies depending on the type of material. As pointed out by the reviewer, the effect of the material of the frequently touched surface with human-skin warming on bacterial survival are important issues to be verified.

**To Reviewer 4:**

**Comment 1:**

Abstract: Please state some actual numbers. Instead of "significantly", state p-value.

**Response:** According to the comment, statistical results have been added together with *p*-values in the abstract of the revised manuscript.

**Comment 2:**

As far as I know, 70% ethanol remains among the most commonly used cleaning agents, but is never mentioned in this manus. I suggest the others make some mention of ethanol for completeness.

**Response:** As pointed out by the reviewer, alcohol is effective in disinfecting high-touch surfaces. However, in order to apply the disinfection measures, it is necessary to periodically clean with alcohol, which takes time and effort. On the other hand, if frequently-touched surfaces can be kept human skin continuously, it will be possible to save time and effort in cleaning work, which may contribute to improving the quality of medical care by reducing work in busy medical settings. We added such explanation about the significance of the human-skin warming effect in the Discussion of the revised manuscript with references.

**Comment 3:**

Methods and Figs 3 and 4, Temperature throughout was very hard to understand. Speculating on what makes sense to me at line 376, I think you are missing the word "incubator". A thermo-hygrostat is only an electronic device used to control the environment in things like incubators. The following reflects my understanding. Hopefully it helps the authors to understand why this was hard to follow and how to revise. "thermo-hygrostat incubator adjusted..."

**Response:** We apologize for the lack of accuracy in the description. According to the comment, we have specified that the main experiments were conducted within a thermo-hygrostat incubator, and have reflected this in the text and figure legends.

**Comment 4:**

Then, at line380, an adjacent handrail is mentioned. Maybe something like this: "approximately 37C when inside the incubator, as measured with FLIR.

**Response:** Very sorry. That's right. We apologize for not describing it correctly. According to the comment, we corrected it.

**Comment 5:**

This now takes us back to line 377. Maybe something like this: "There were 2 adjacent handrails; temperatures were monitored..."

**Response:** Very sorry. As pointed put by the reviewer, one handrail was used as a control without heating, and the other was heated with a heater. According to the comment, we corrected it.

**Comment 6:**

Some clarification is needed as to the differences in how the different handrails were used.

**Response:** As already mentioned, we set up two handrails inside the thermo-hygrostat incubator and conducted experiments. Also, at that time, one handrail was used as a control without a heater, and the other was heated to verify the human-skin warming effect. We have revised the description of the method to reflect this. Again, we apologize for the lack of explanations related to the experimental handrail devise with heater.
